# Supplementary figures and images for: Destabilizers of the thymidylate synthase homodimer accelerate its proteasomal degradation and inhibit cancer growth
Source: eLife. 2022 Dec 7;11:e73862. doi: 10.7554/eLife.73862 (PMC9831607; doi:10.7554/eLife.73862)

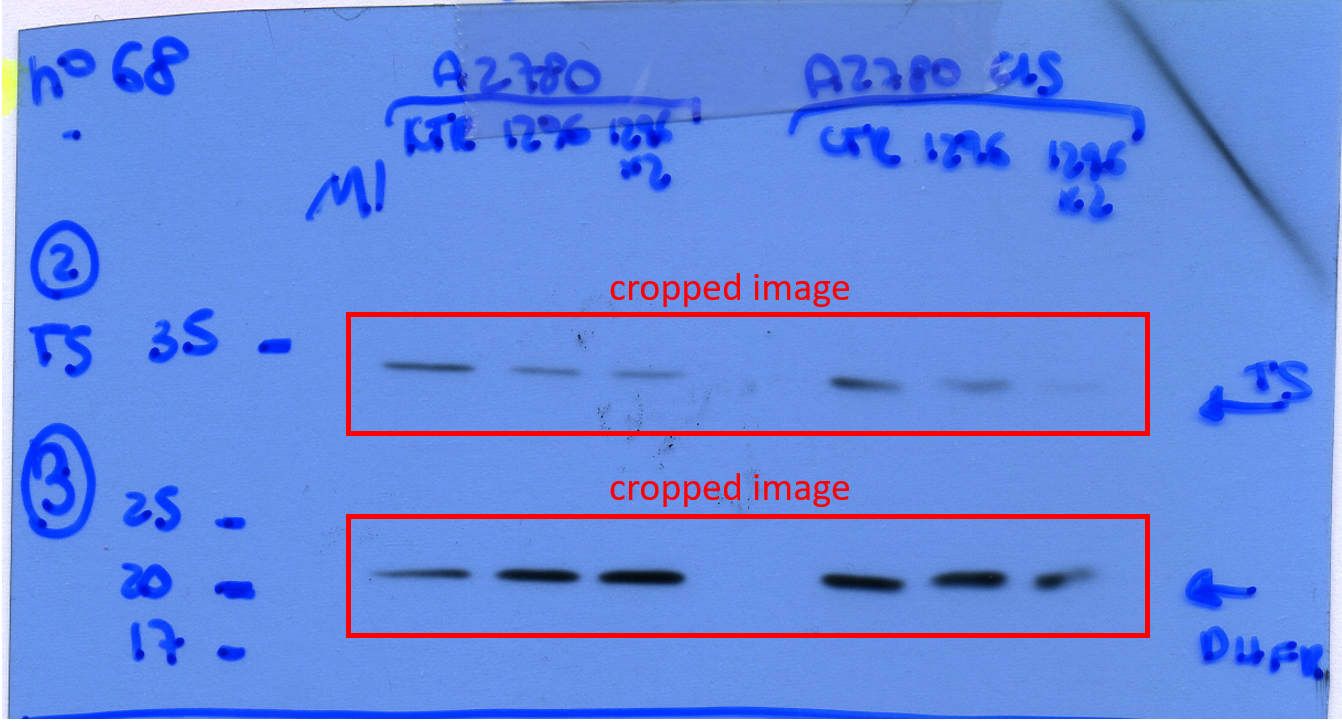

Supplement: Figure 7—source data 1. [file elife-73862-fig7-data1.zip › Figure 7-Source data 1_ left TS-DHFR.PNG]

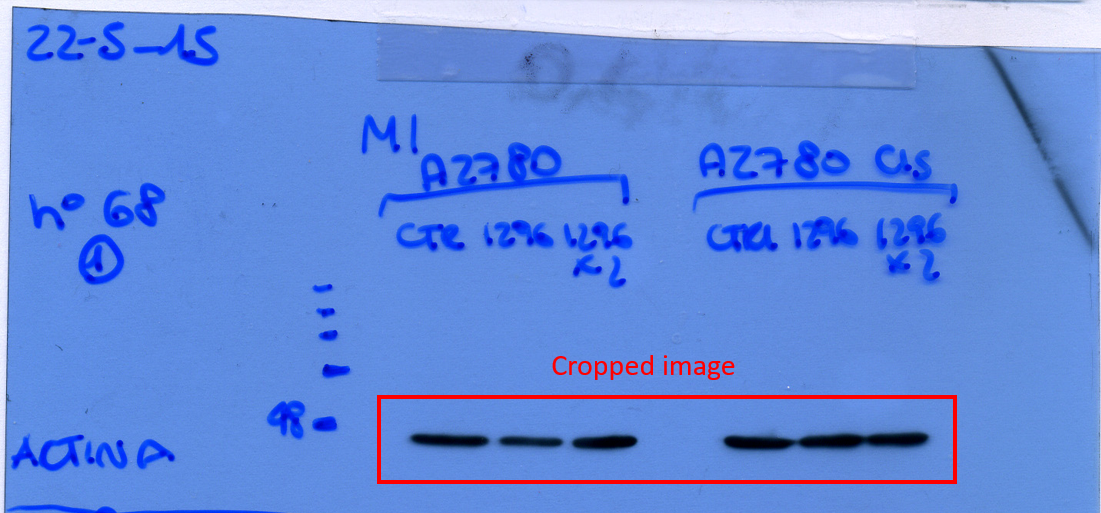

Supplement: Figure 7—source data 2. [file elife-73862-fig7-data2.zip › Figure 7-Source data 2_ left actin.PNG]

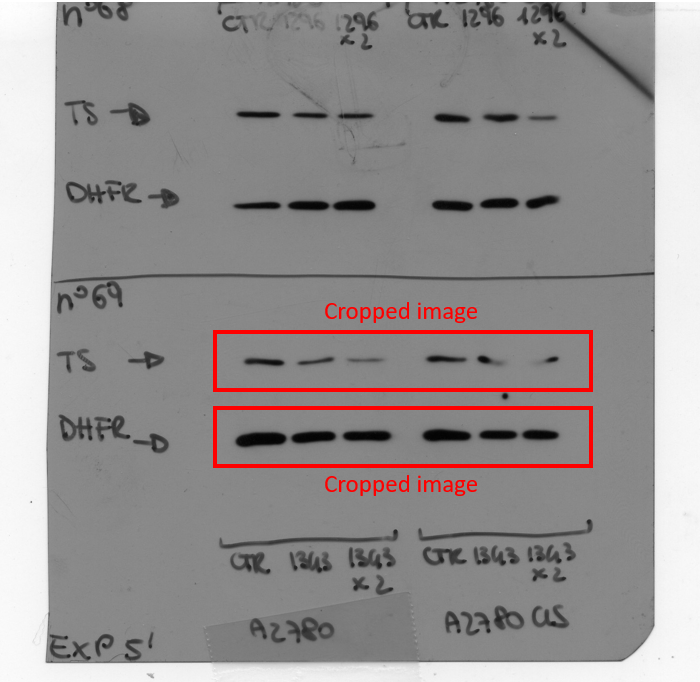

Supplement: Figure 7—source data 3. [file elife-73862-fig7-data3.zip › Figure 7-Source data 3_right E7_A2780-CP-TS-DHFR.png]

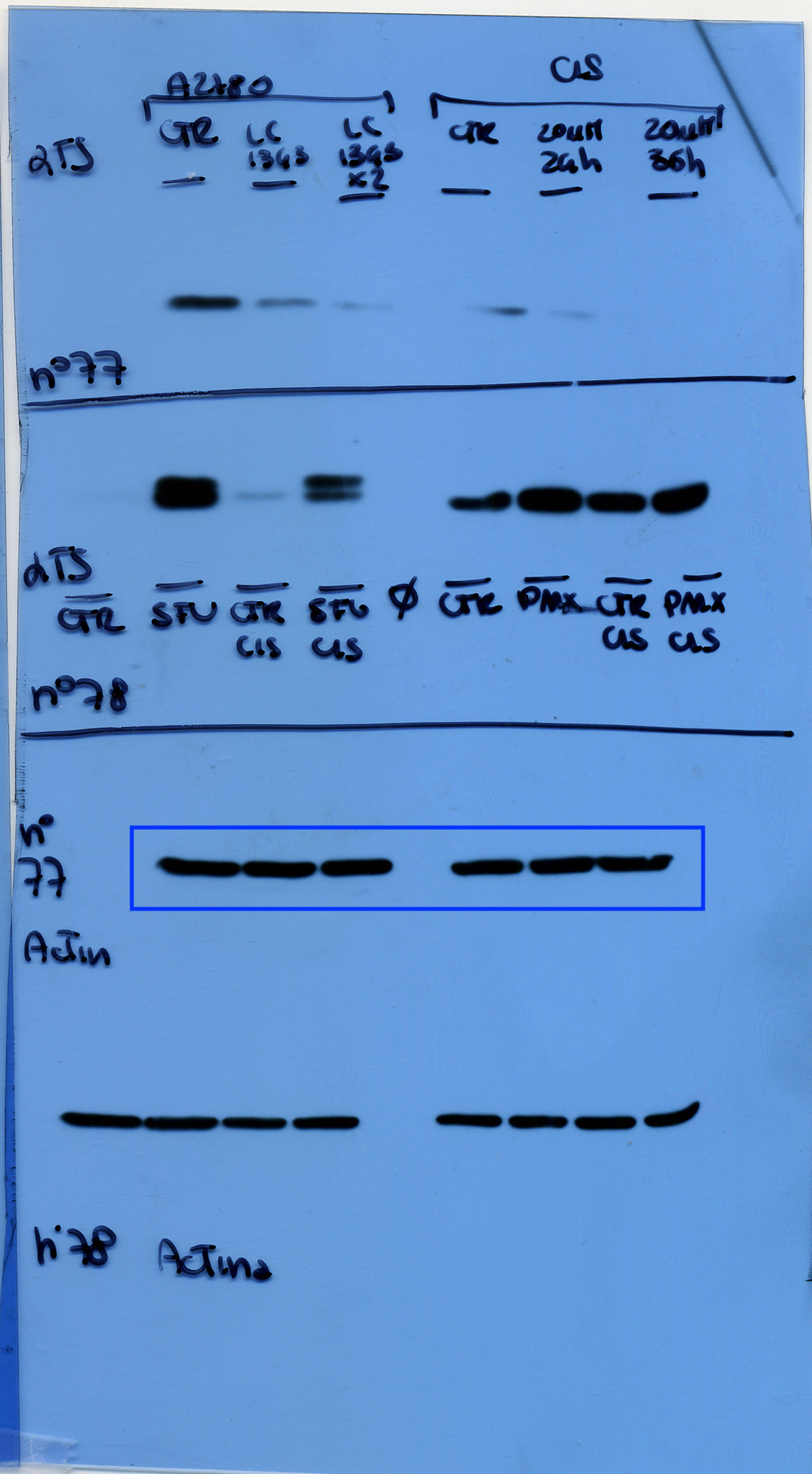

Supplement: Figure 7—source data 4. [file elife-73862-fig7-data4.zip › Figure7A-Source data 4actin.jpg]

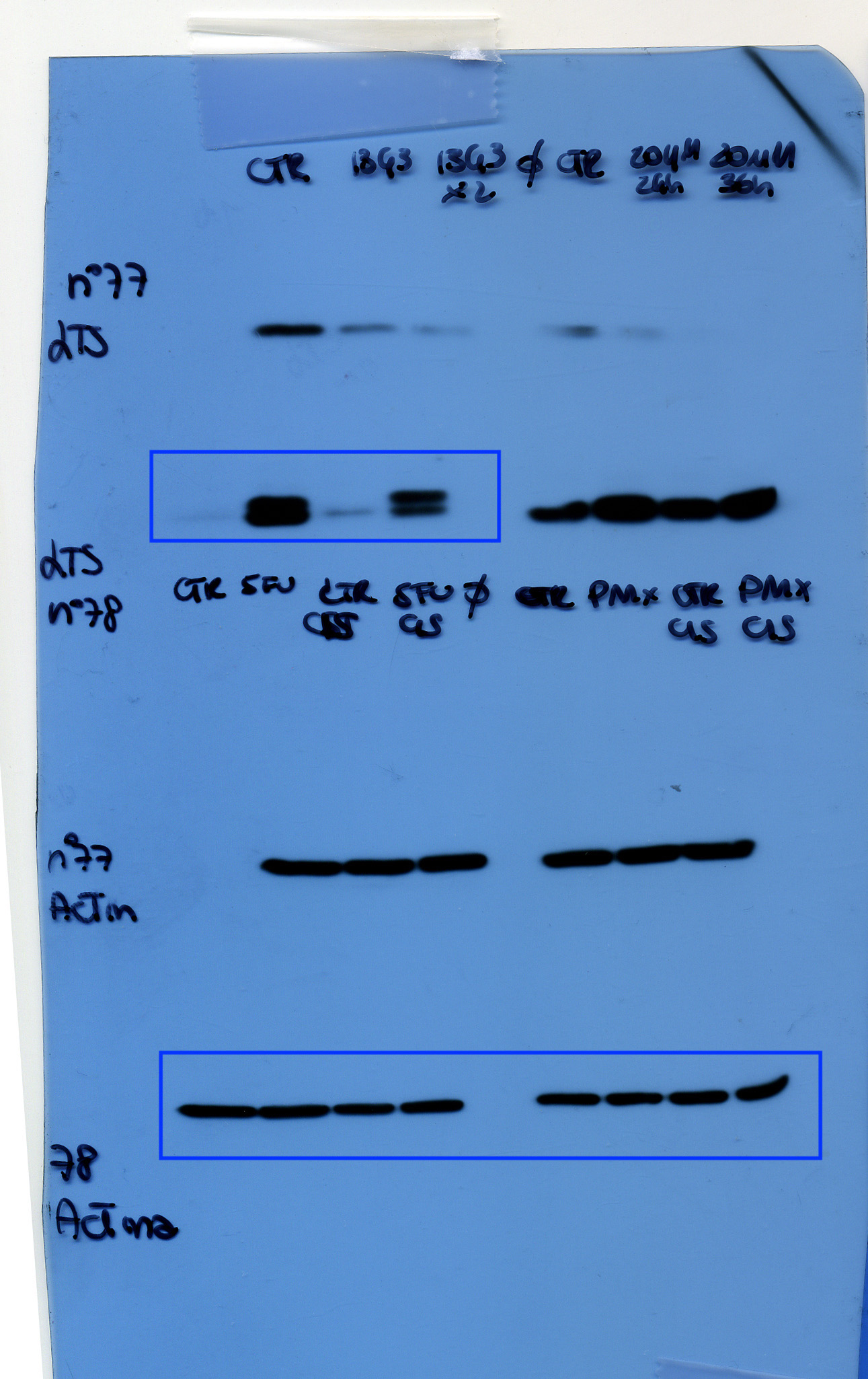

Supplement: Figure 7—source data 5. [file elife-73862-fig7-data5.zip › Figure 7-Source data_5.jpg]

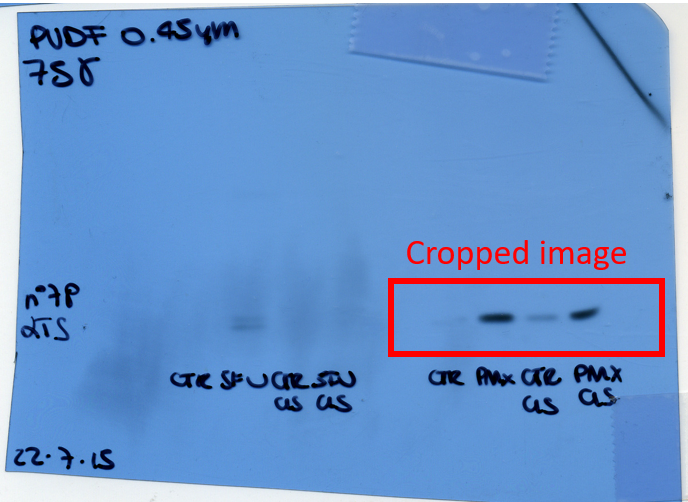

Supplement: Figure 7—source data 6. [file elife-73862-fig7-data6.zip › Figure 7-Source data 7. Left PMX.png]

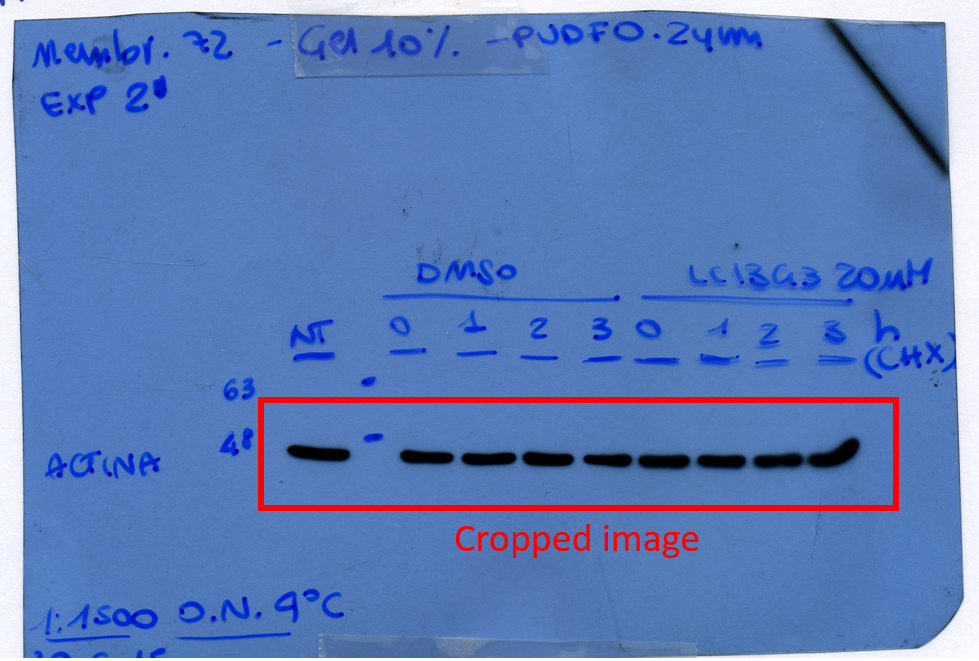

Supplement: Figure 7—source data 7. [file elife-73862-fig7-data7.zip › Figure 7-Source data 7 .png]

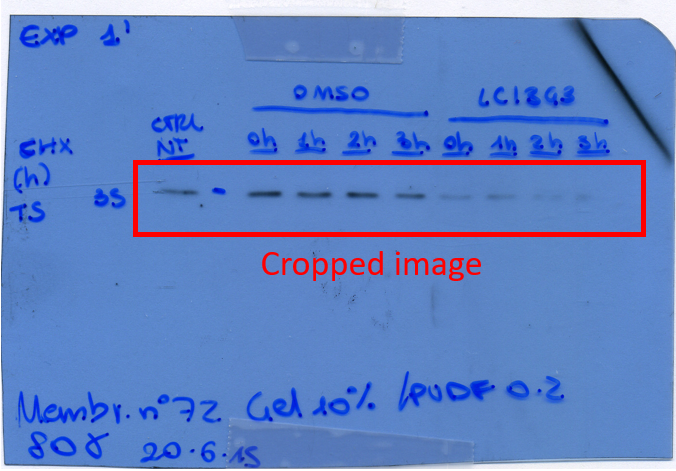

Supplement: Figure 7—source data 8. [file elife-73862-fig7-data8.zip › Figure 7-Source data 8. .png]

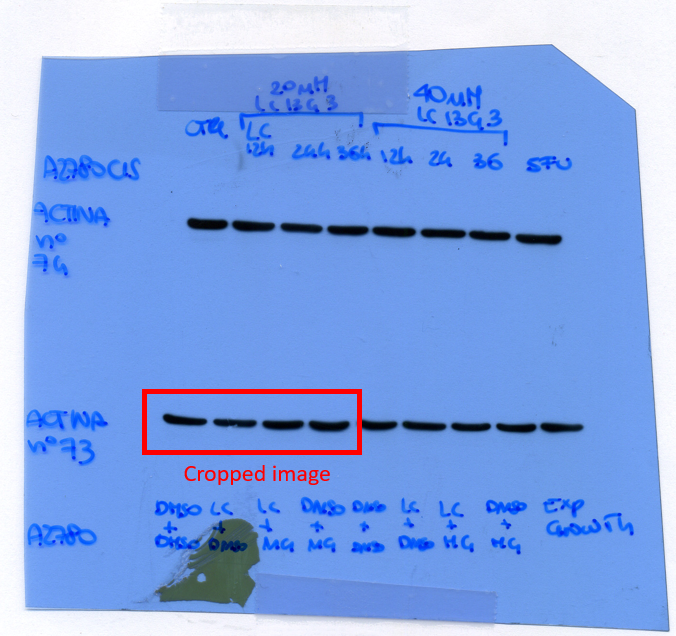

Supplement: Figure 7—source data 9. [file elife-73862-fig7-data9.zip › Figure 7-Source data 9.png]

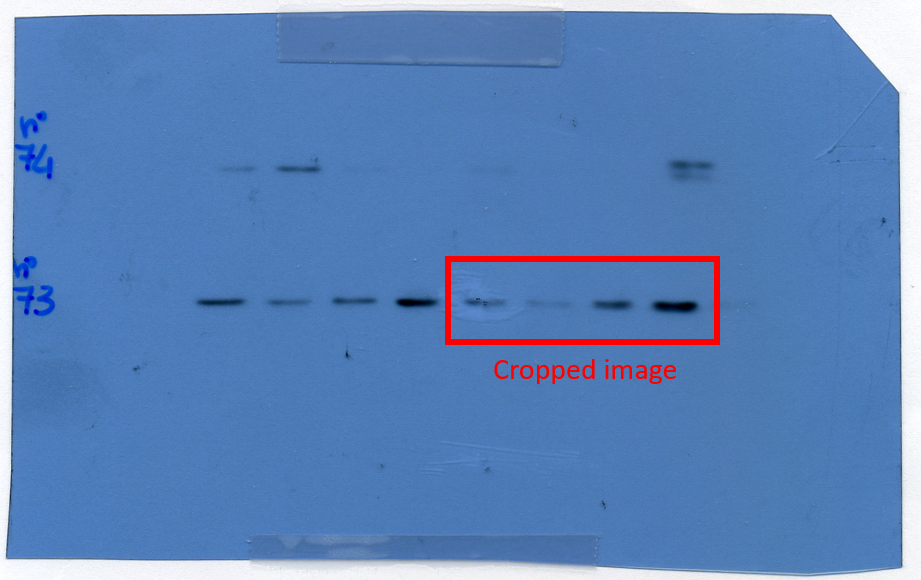

Supplement: Figure 7—source data 10. [file elife-73862-fig7-data10.zip › Figure 7-Source data 10.png]

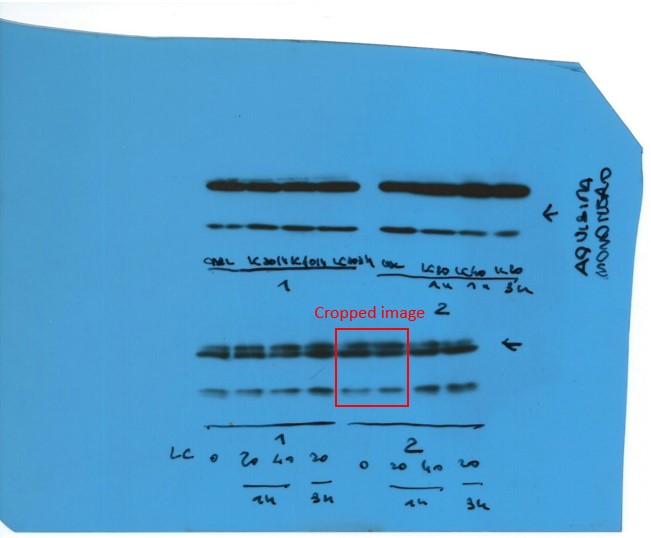

Supplement: Figure 7—source data 11. [file elife-73862-fig7-data11.zip › Figure 7-Source data 12. Dimer-monomer-E7-TS.jpg]

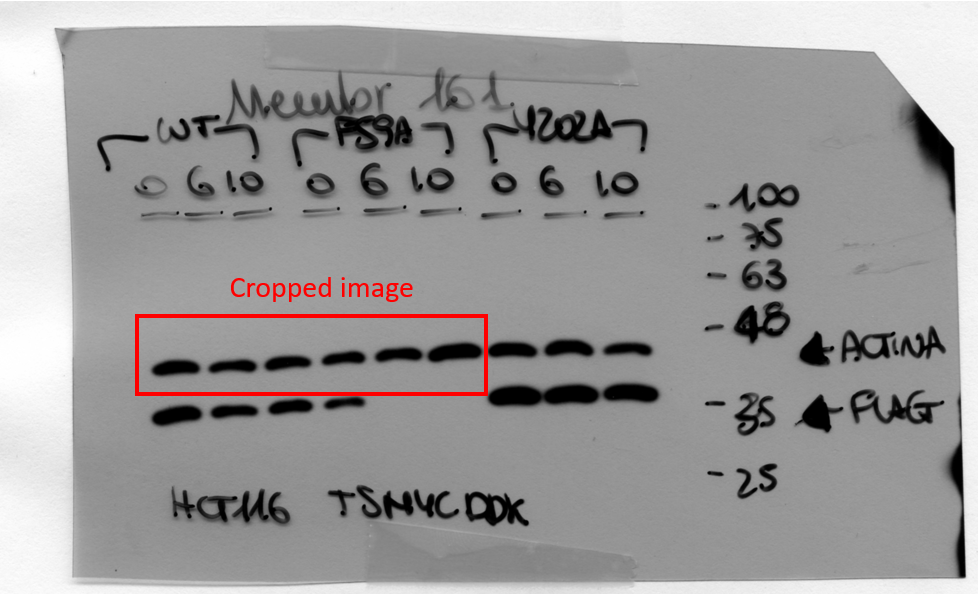

Supplement: Figure 7—source data 13. [file elife-73862-fig7-data13.zip › Figure 7-Source data 13.png]

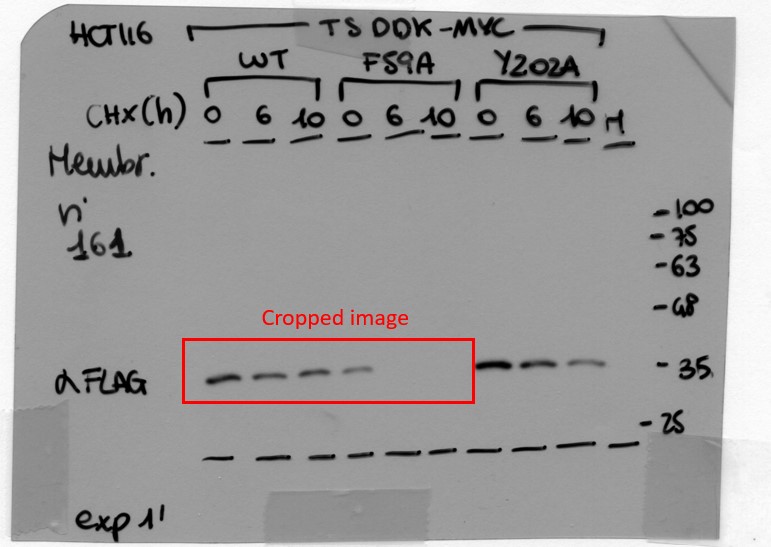

Supplement: Figure 7—source data 14. [file elife-73862-fig7-data14.zip › Figure 7-Source data 14.jpg]

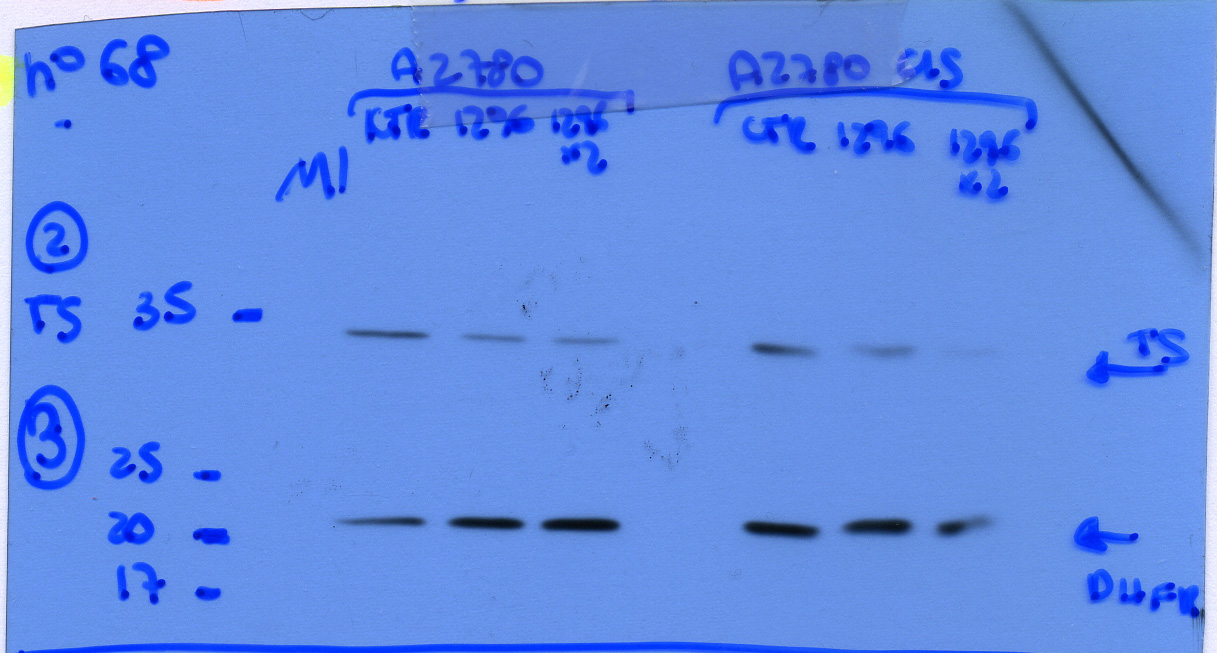

Supplement: Figure 7—source data 15. [file elife-73862-fig7-data15.zip › Figure 7-Source data 15.jpg]

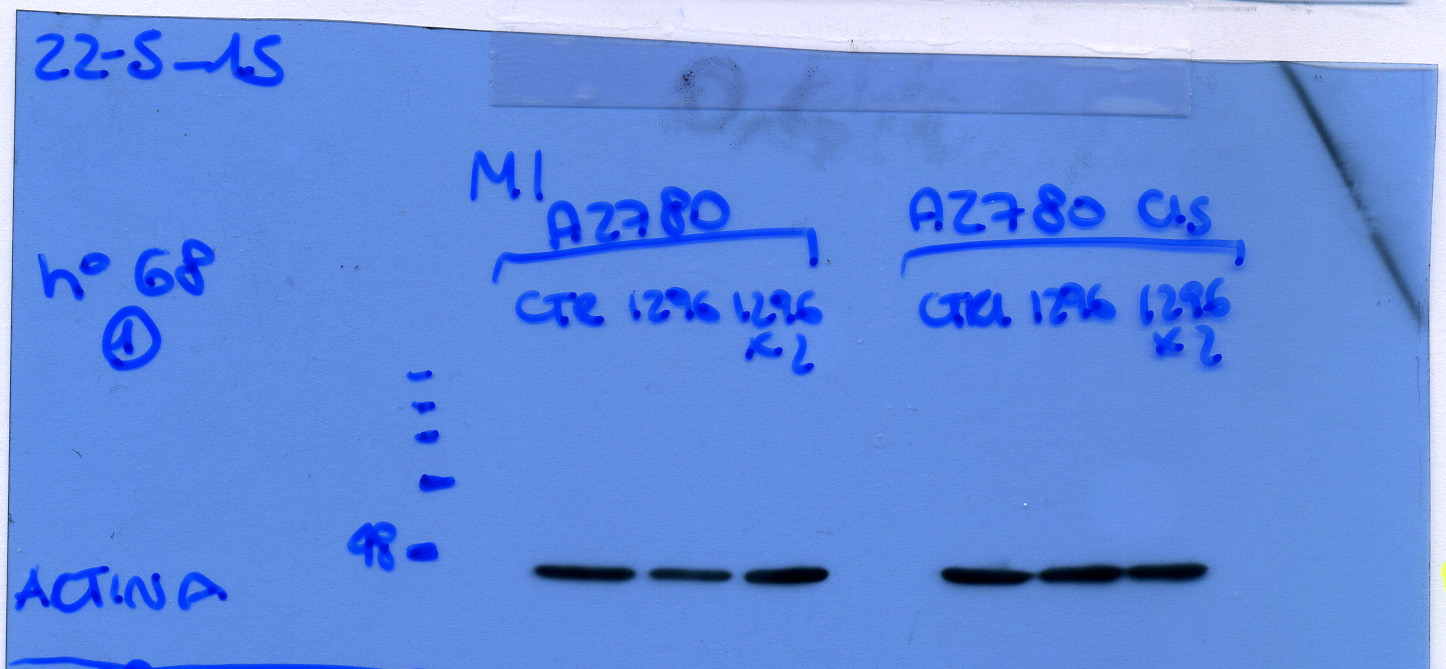

Supplement: Figure 7—source data 16. [file elife-73862-fig7-data16.zip › Figure7_Source data 16.jpg]

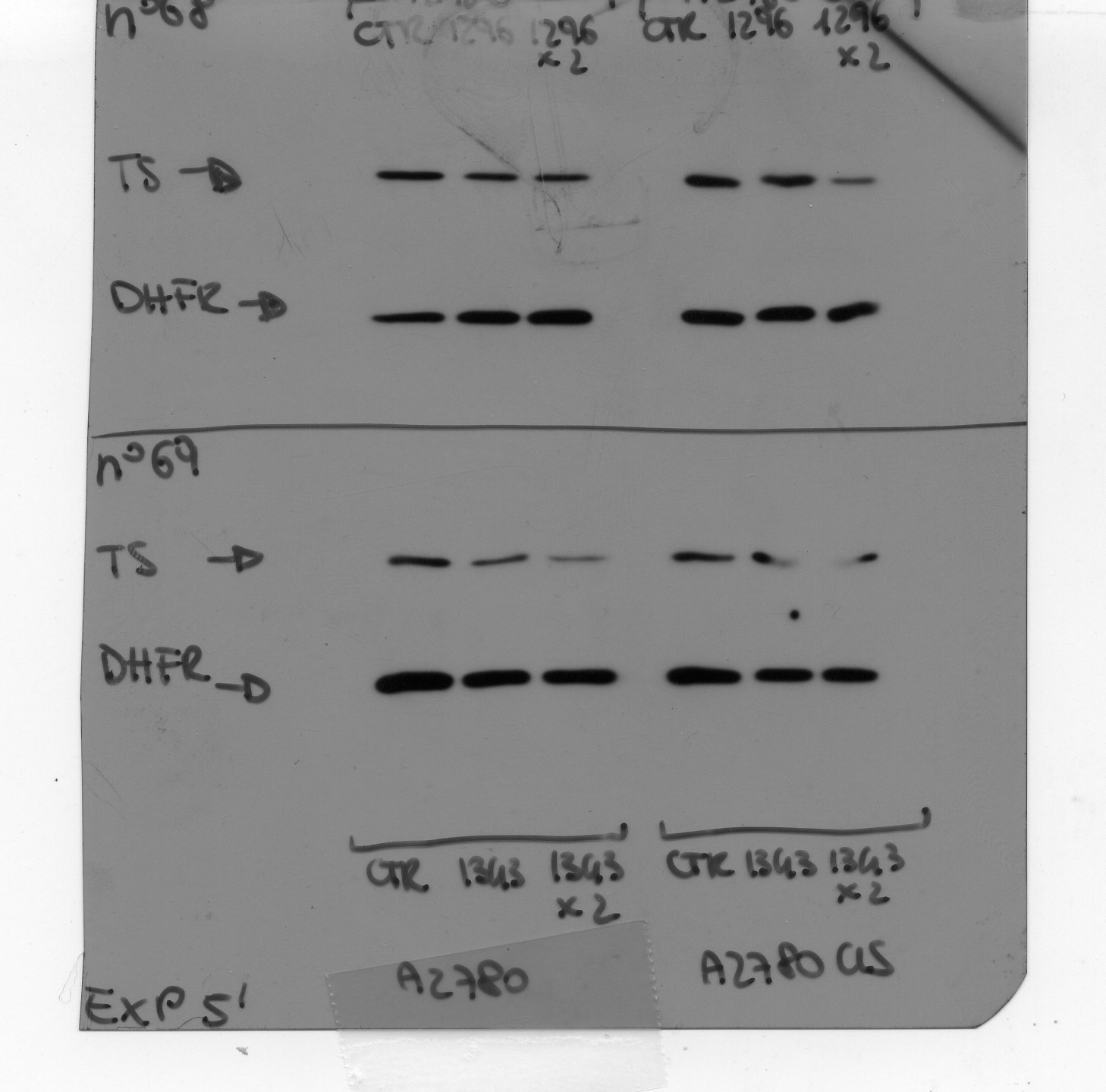

Supplement: Figure 7—source data 17. [file elife-73862-fig7-data17.zip › Figure7_Source data 17.jpg]

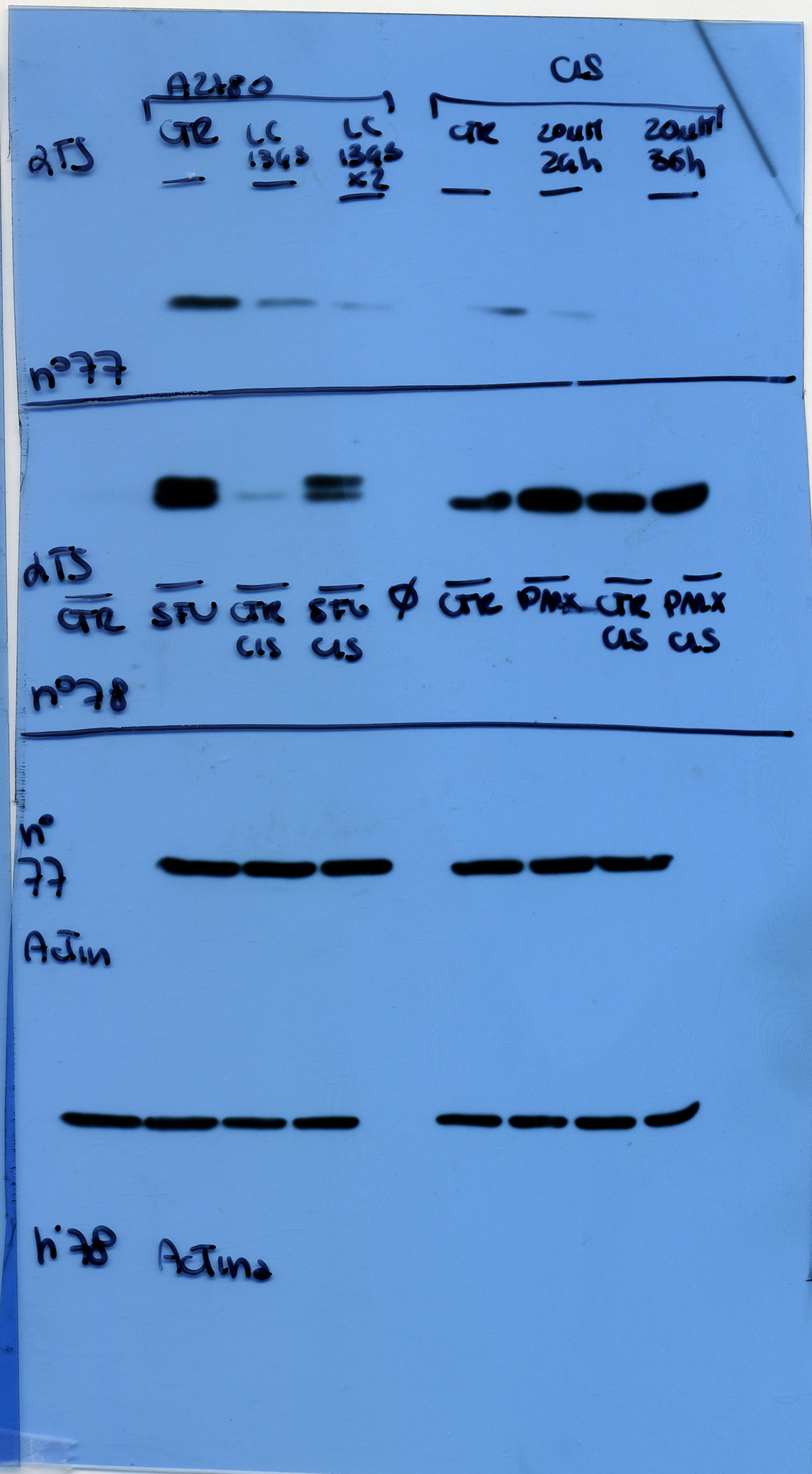

Supplement: Figure 7—source data 18. [file elife-73862-fig7-data18.zip › Figure7-Source data 18.jpg]

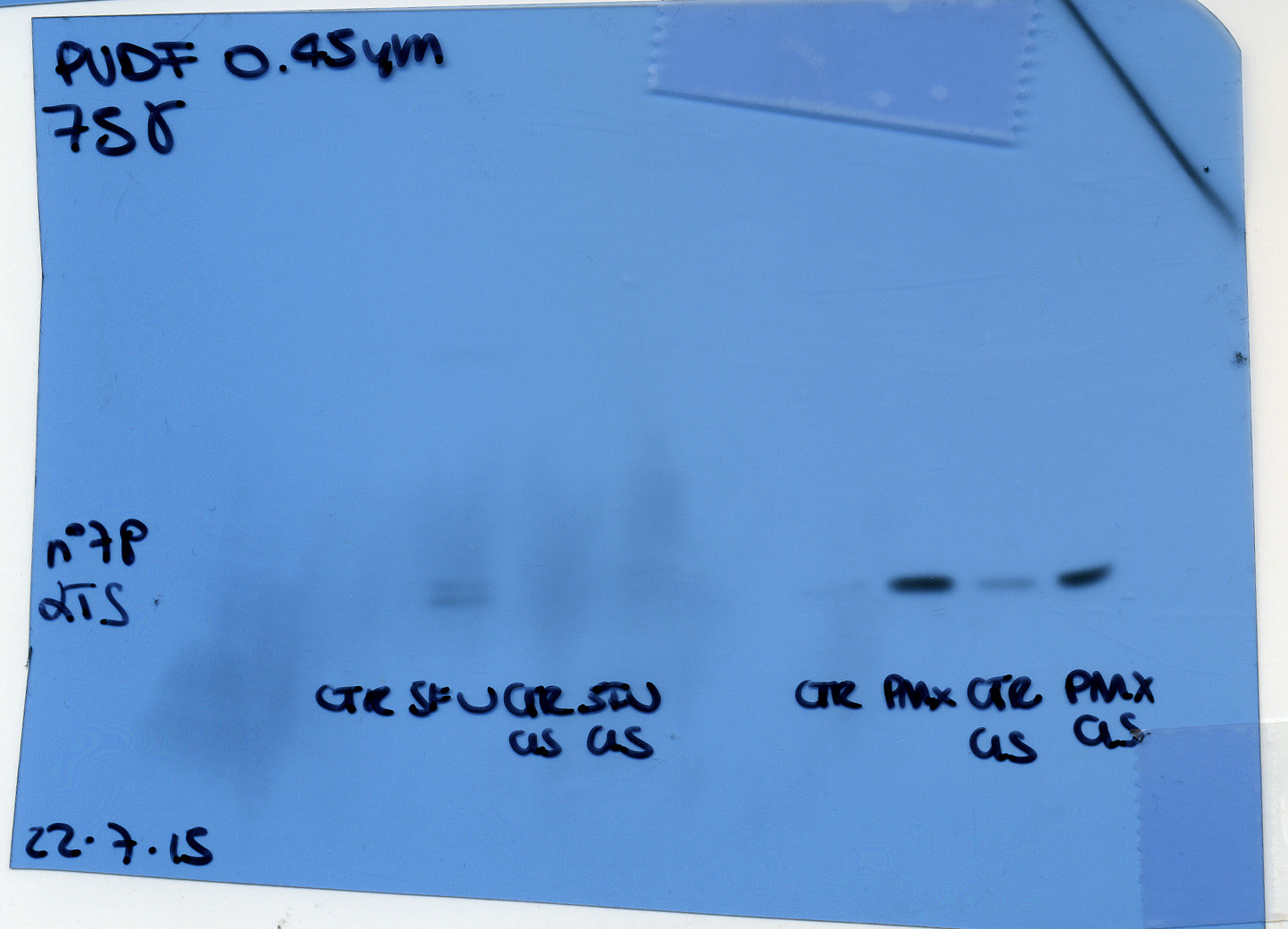

Supplement: Figure 7—source data 20. [file elife-73862-fig7-data20.zip › Figure7-Source data 20.jpg]

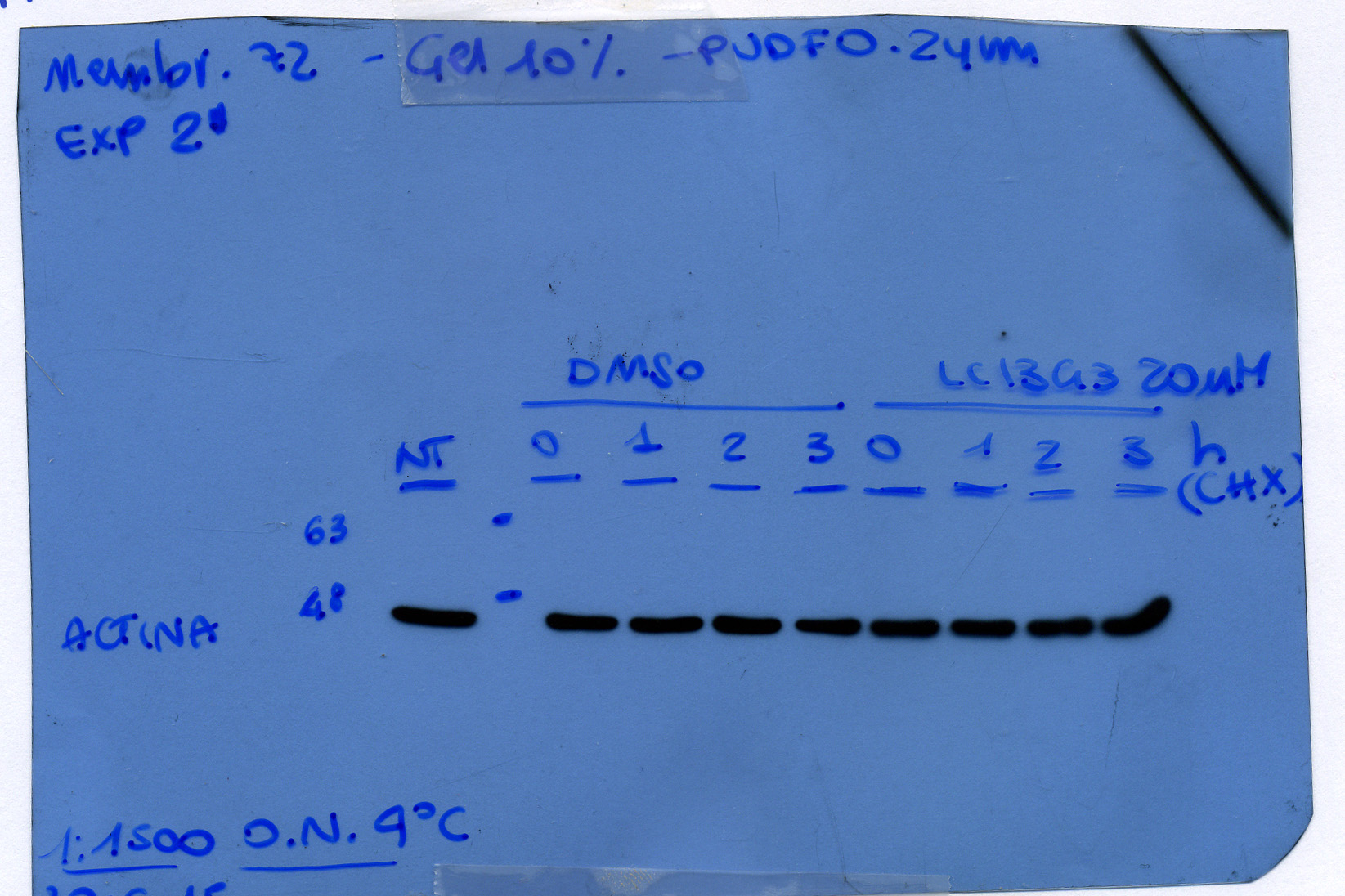

Supplement: Figure 7—source data 21. [file elife-73862-fig7-data21.zip › Figure7-Source data 21.jpg]

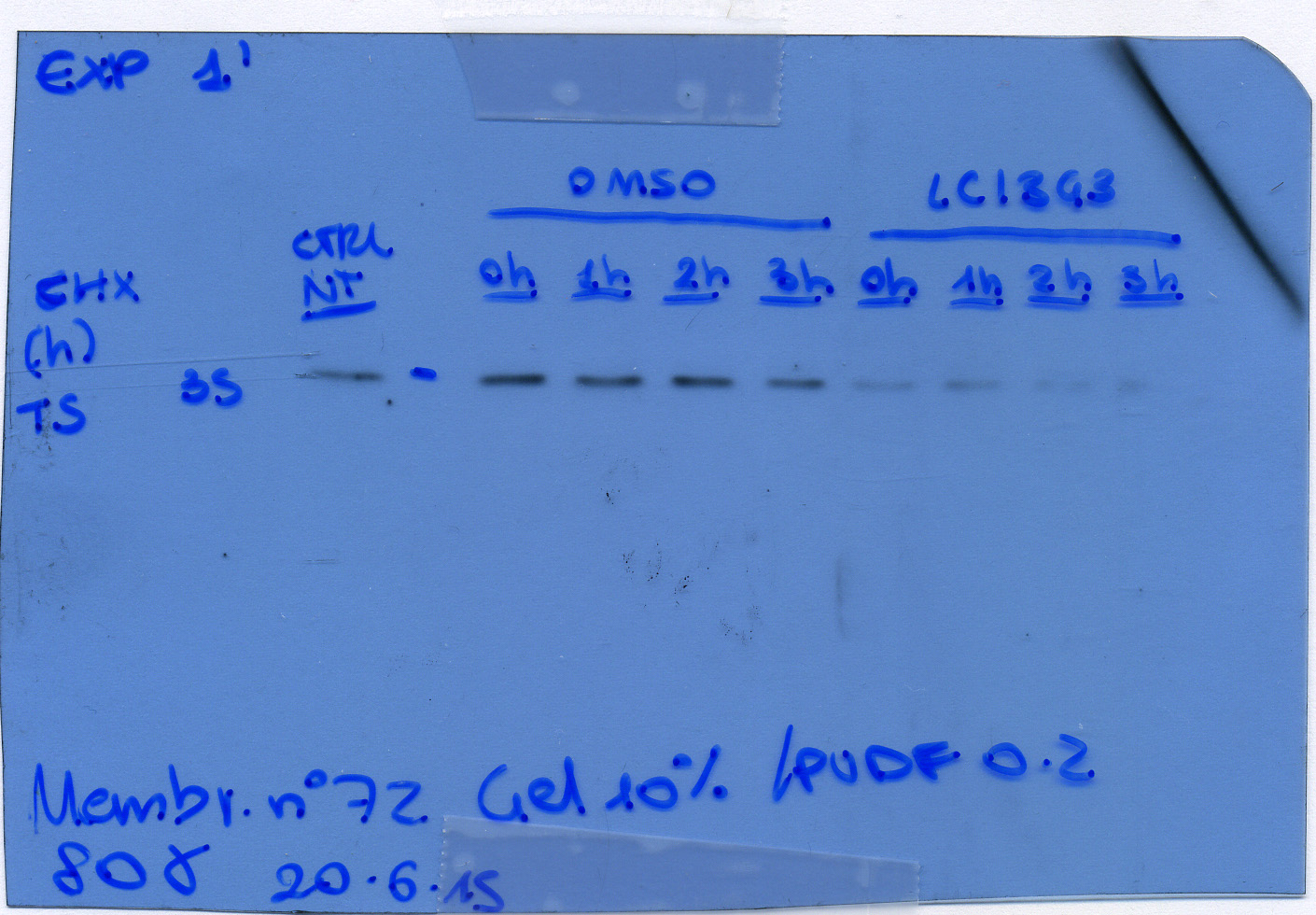

Supplement: Figure 7—source data 22. [file elife-73862-fig7-data22.zip › Figure7-Source data 22.jpg]

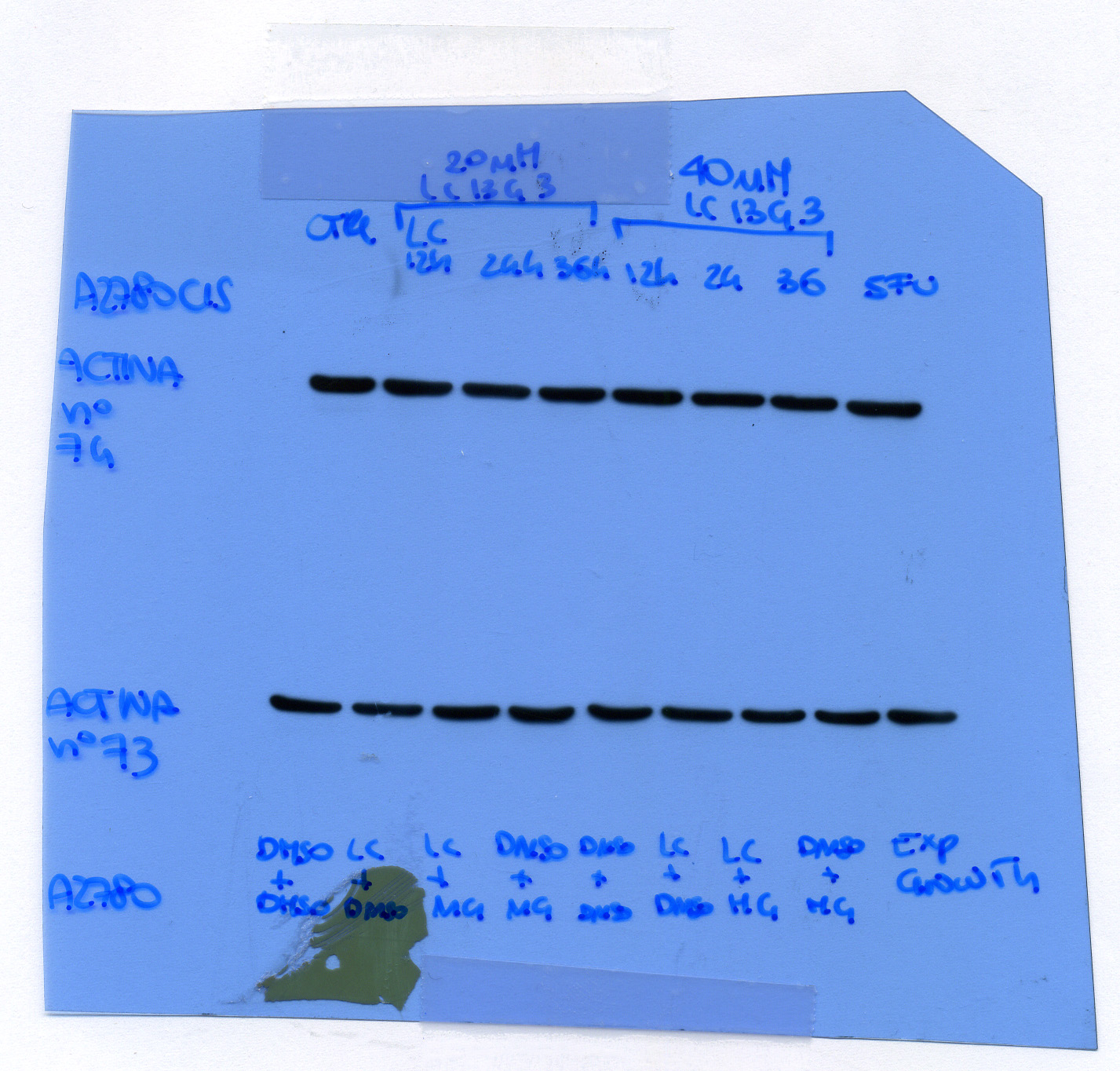

Supplement: Figure 7—source data 23. [file elife-73862-fig7-data23.zip › Figure7-Source data 23.jpg]

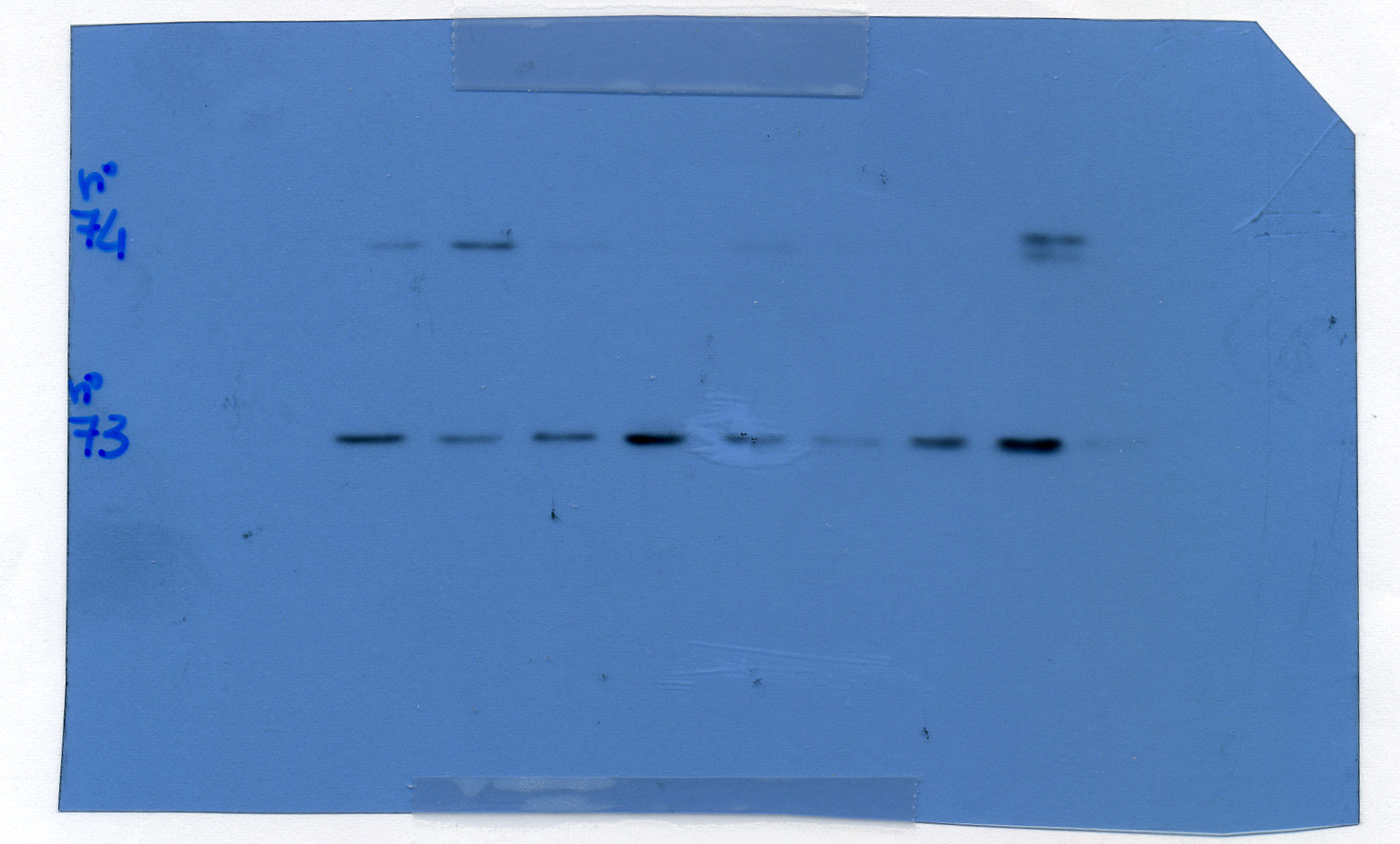

Supplement: Figure 7—source data 24. [file elife-73862-fig7-data24.zip › Figure7-Source data 24.jpg]

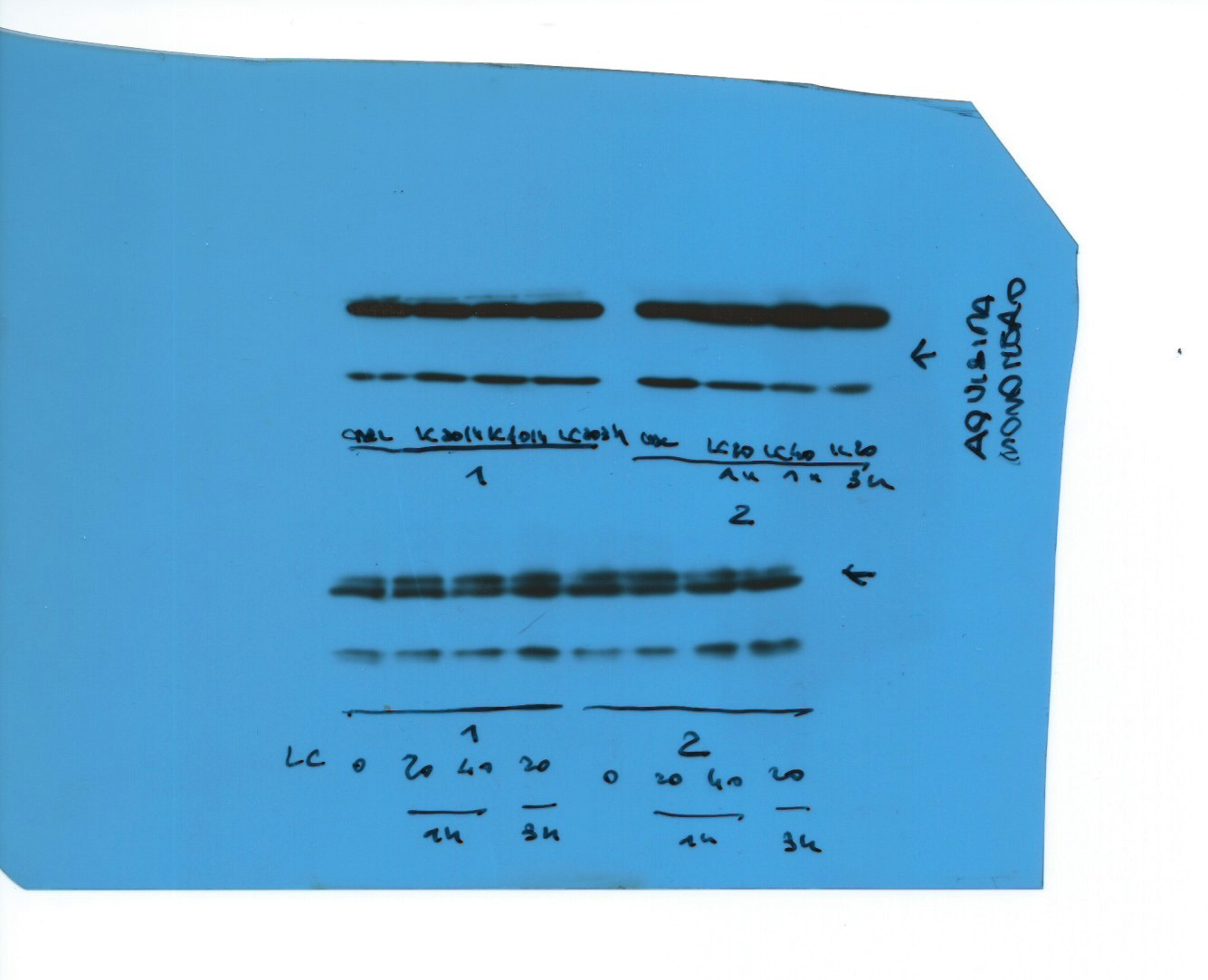

Supplement: Figure 7—source data 25. [file elife-73862-fig7-data25.zip › Figure 7- Source data 25. .jpg]

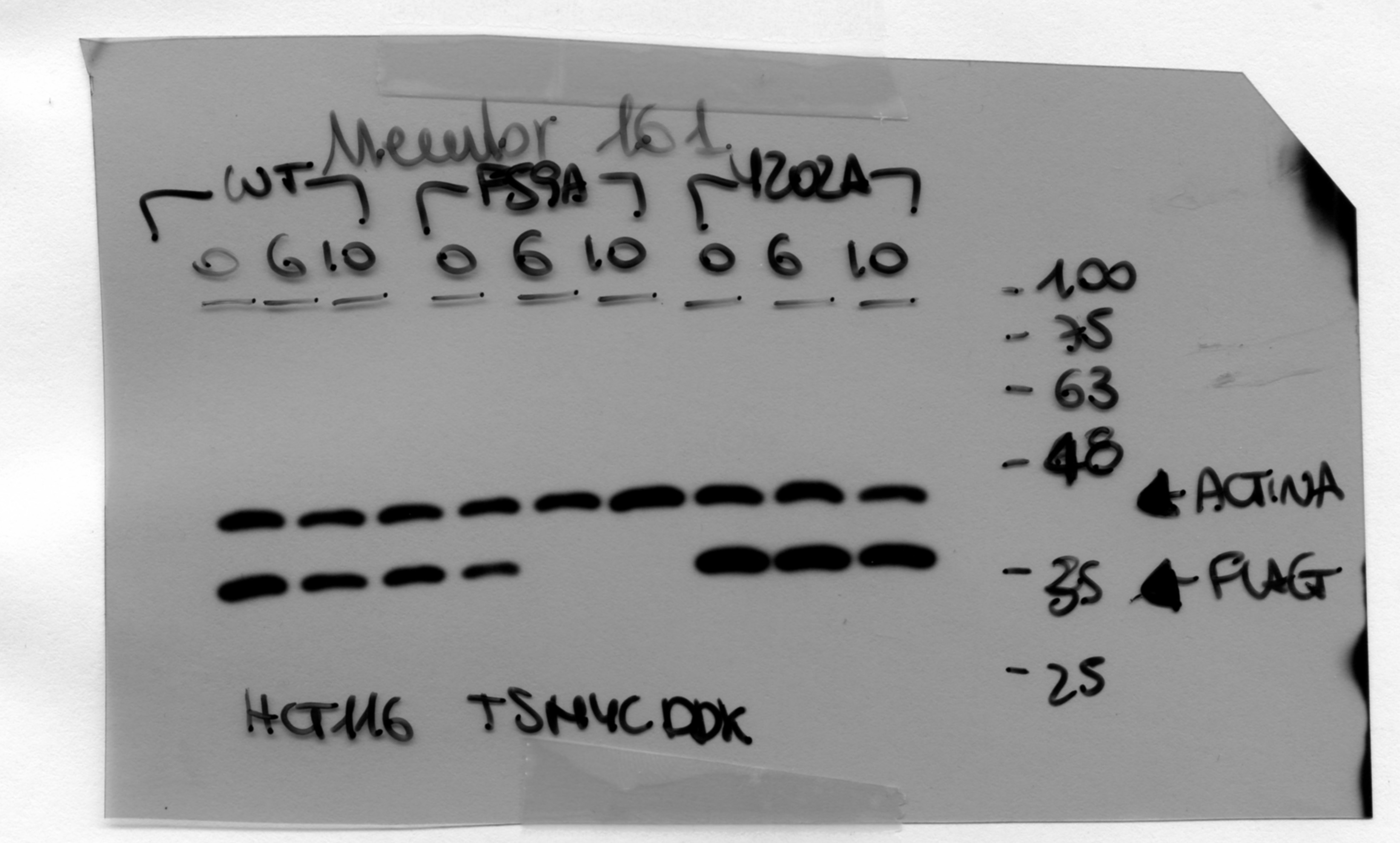

Supplement: Figure 7—source data 26. [file elife-73862-fig7-data26.zip › Figure 7 - Source data 26.jpg]

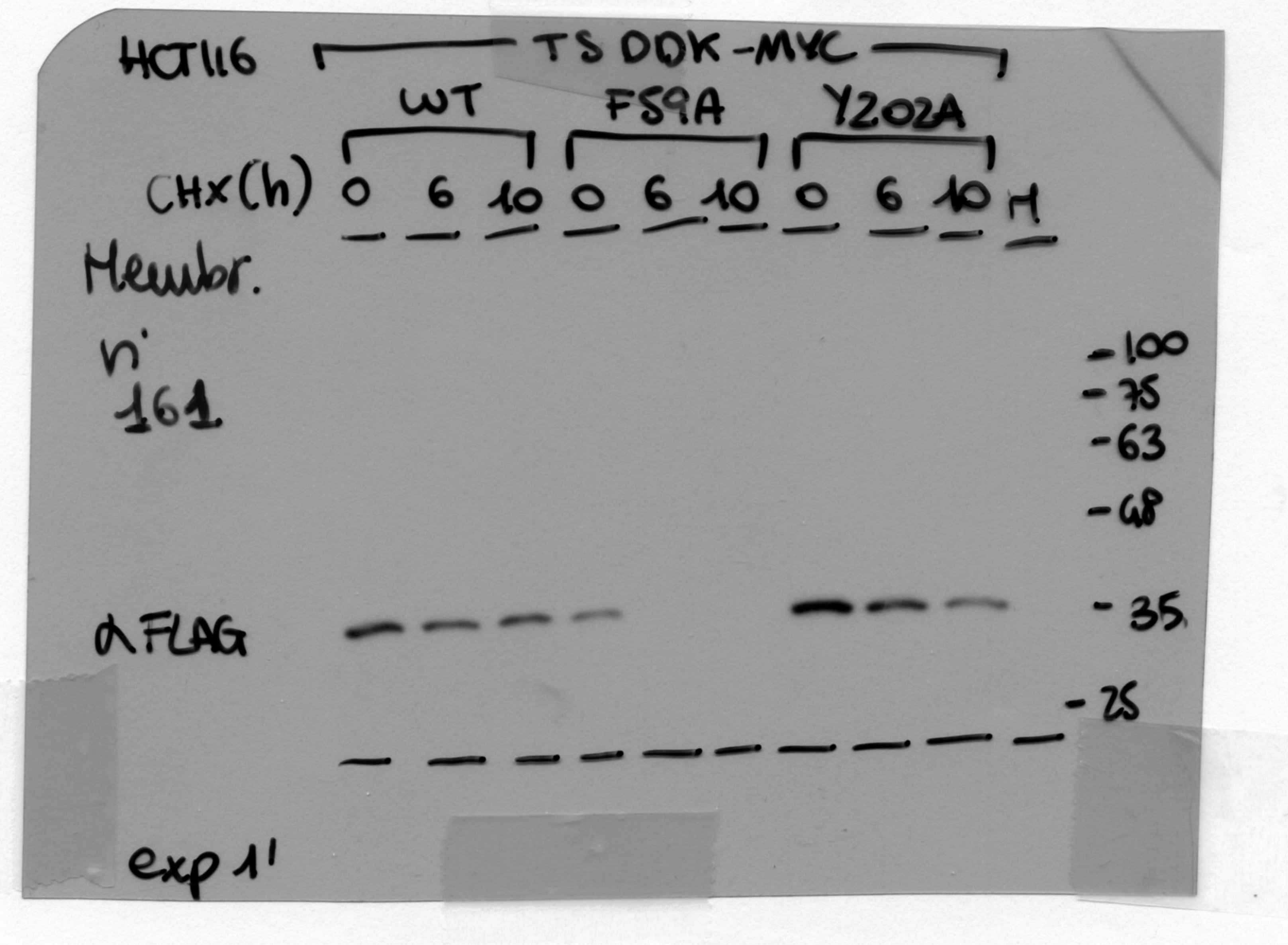

Supplement: Figure 7—source data 27. [file elife-73862-fig7-data27.zip › Figure7-Source data 27.jpg]

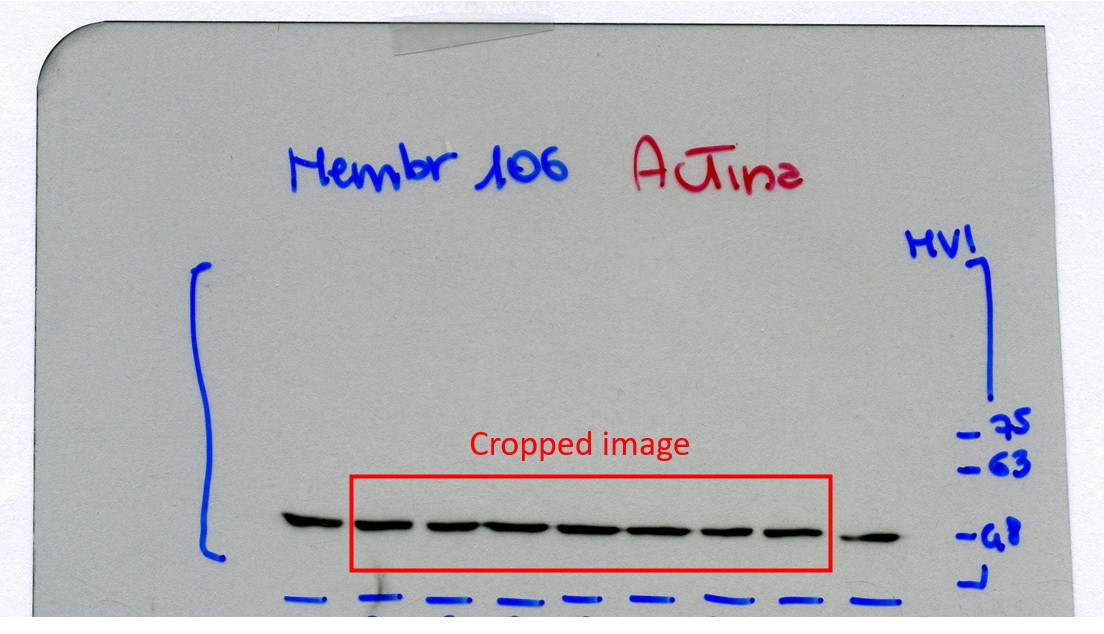

Supplement: Figure 7—figure supplement 1—source data 2. [file elife-73862-fig7-figsupp1-data2.zip › Figure 7-figure supplementary 1-Source data 2. E7_HT29- Actin .jpg]

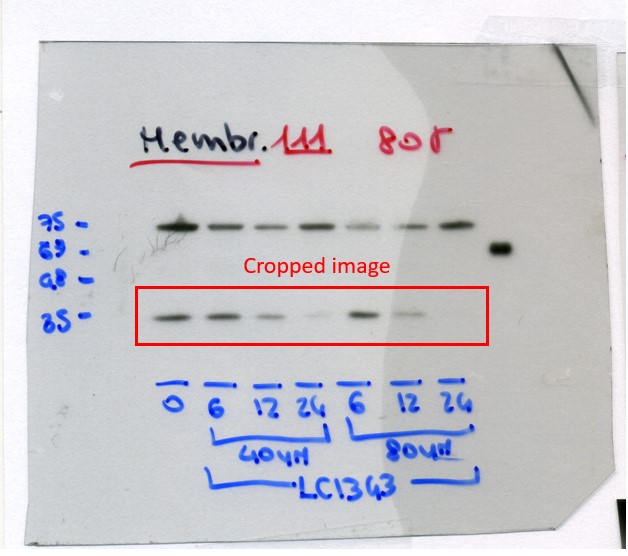

Supplement: Figure 7—figure supplement 1—source data 3. [file elife-73862-fig7-figsupp1-data3.zip › Figure 7-figure supplementary 1-Source data 3.jpg]

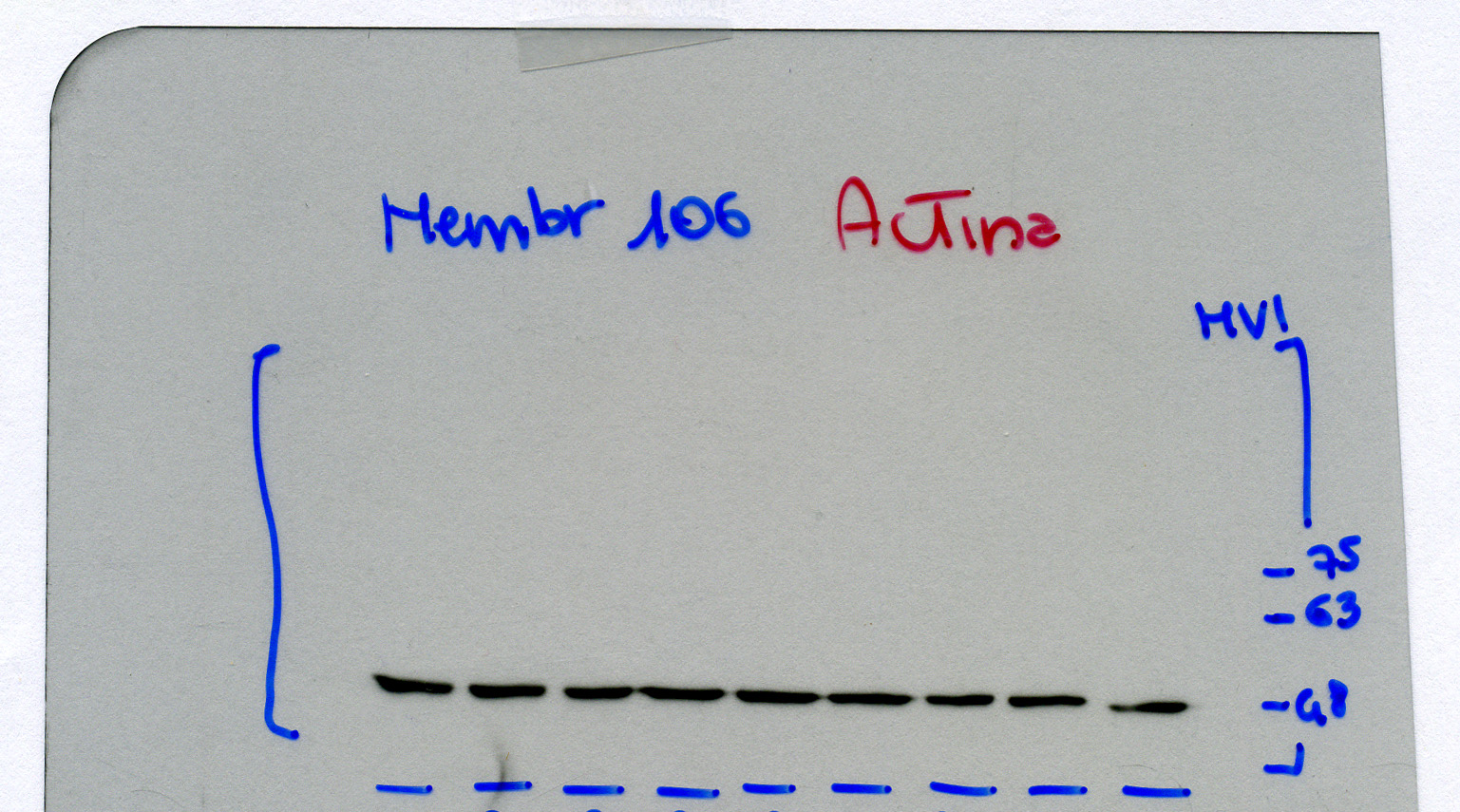

Supplement: Figure 7—figure supplement 1—source data 4. [file elife-73862-fig7-figsupp1-data4.zip › Figure 7- figure supplement 1C-Source data 1b.-Actina.jpg]

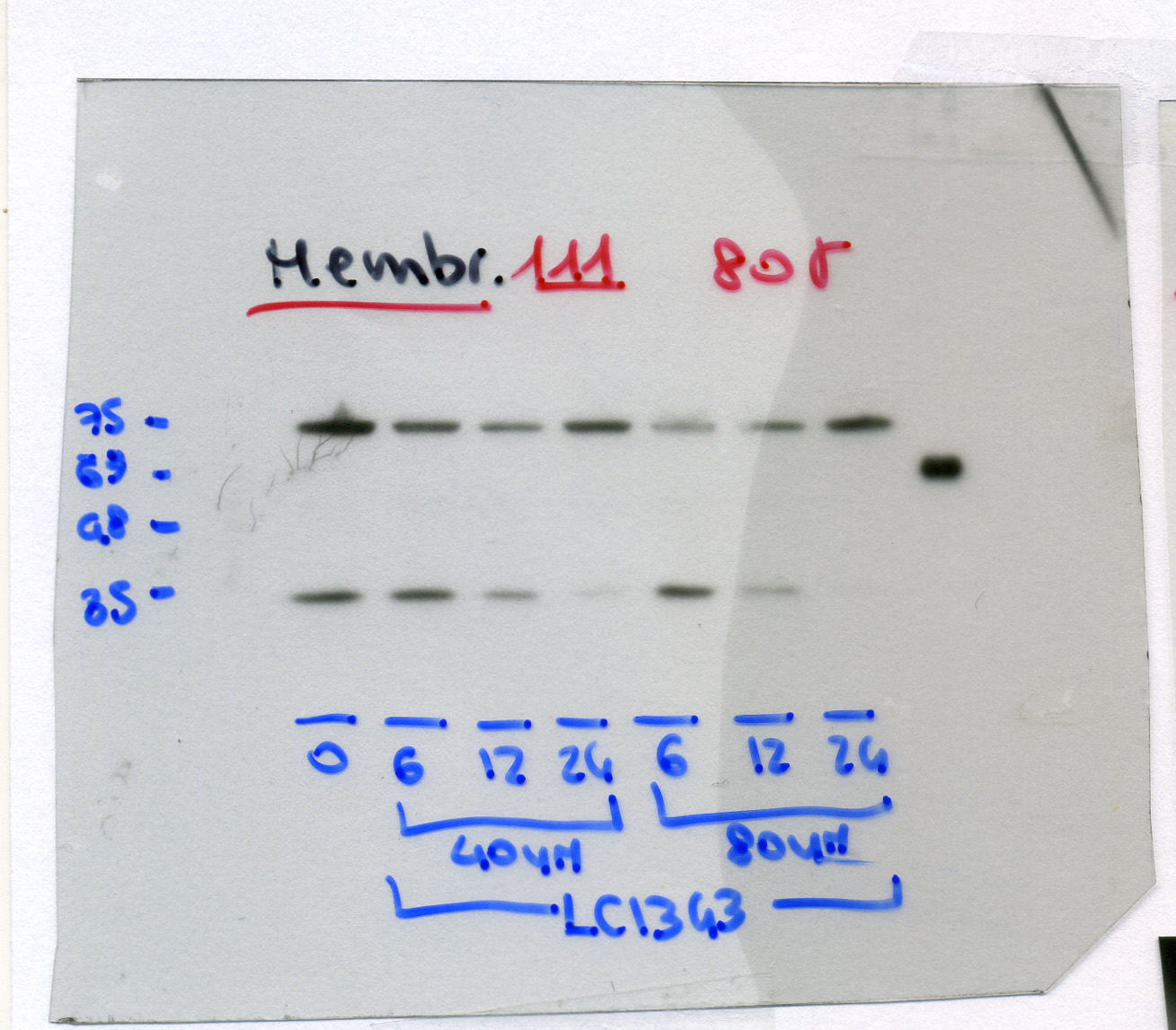

Supplement: Figure 7—figure supplement 1—source data 5. [file elife-73862-fig7-figsupp1-data5.zip › Figure 7- figure supplement 1-Source data 5.jpg]

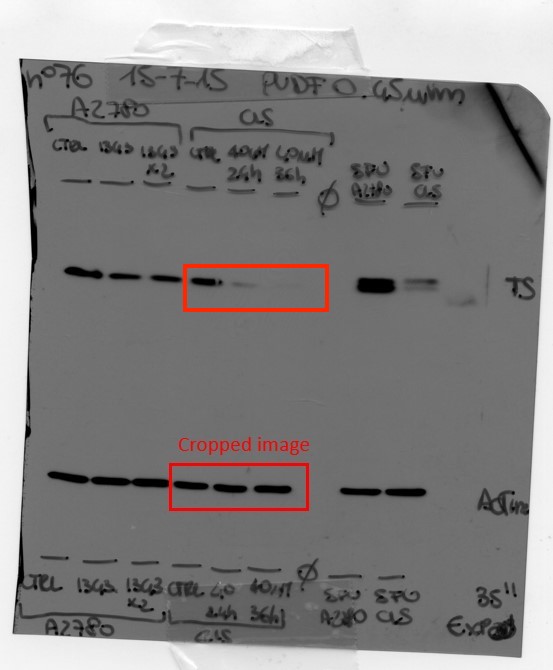

Supplement: Figure 7—figure supplement 1—source data 6. [file elife-73862-fig7-figsupp1-data6.zip › Figure 7-figure supplement 1-Source data 4 .jpg]

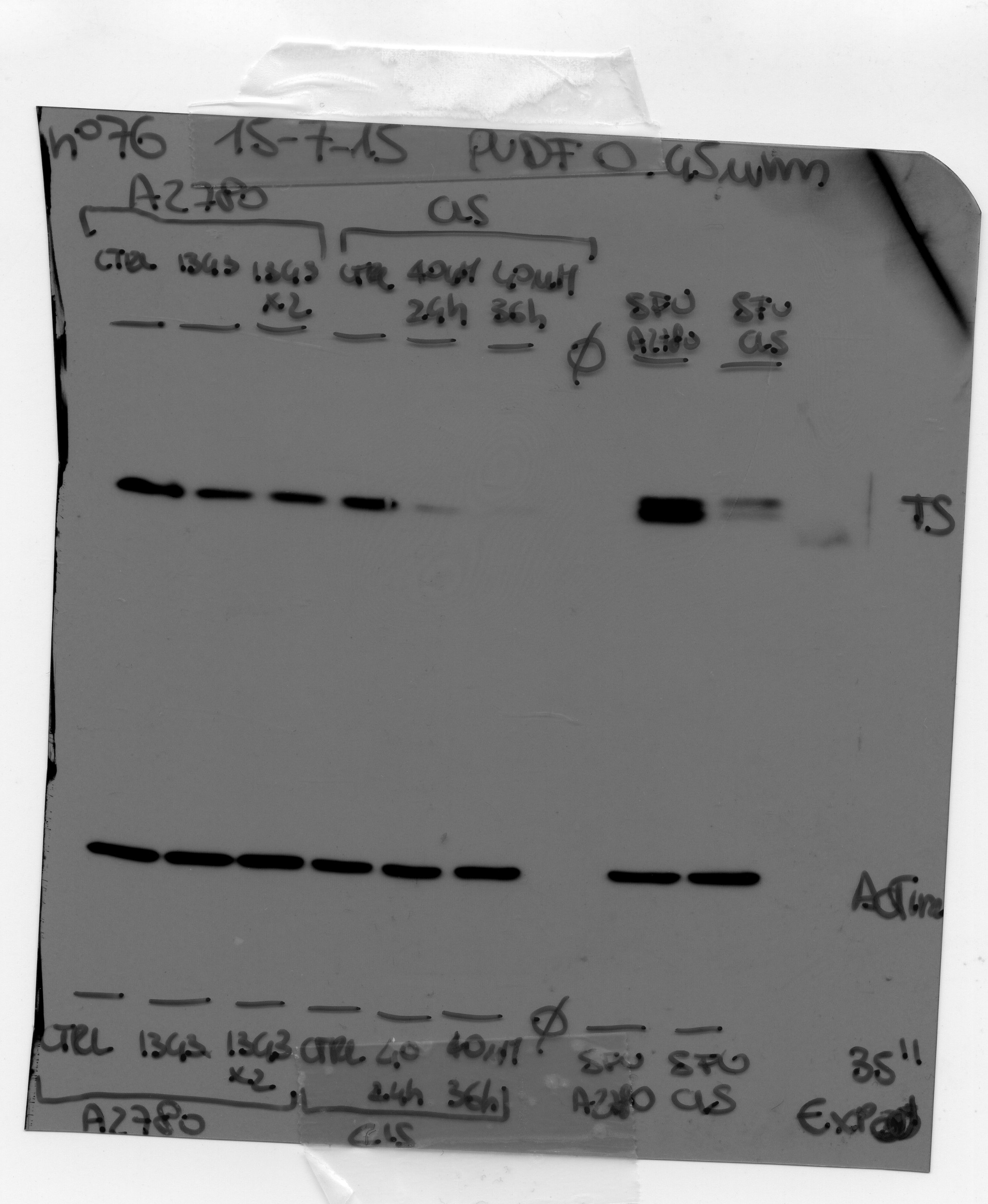

Supplement: Figure 7—figure supplement 1—source data 7. [file elife-73862-fig7-figsupp1-data7.zip › Figure 7-figure supplement 1 - Source data 7.jpg]
