# Supplementary material for: Destabilizers of the thymidylate synthase homodimer accelerate its proteasomal degradation and inhibit cancer growth
Source: eLife. 2022 Dec 7;11:e73862. doi: 10.7554/eLife.73862 (PMC9831607; doi:10.7554/eLife.73862)
Supplement: Figure 7—source data 12. [file elife-73862-fig7-data12.docx]

**Figure 7-source data 12. Effect of Dimer disrupters on hTS levels and half-life in human cancer cell lines. (in Figure C,D,G)**

**Figure 7C-source data 1.** Half-life calculation of hTS protein in A2780 cells exposed to CHX for 0-22h. Data indicate mean values and standard deviation (SD) of biological repeats performed in duplicate.

| **A2780** | TS protein level (%) |
| --- | --- |
| **0h** | 100 |
| **3h** | 45±3 |
| **6h** | 15±7 |
| **9h** | 5±3 |
| **12h** | 0 |
| **22h** | 0 |

**Figure 7D-source data 1.** Stability of hTS protein in A2780 cells treated with E7 for 12 h, then with CHX for 0-3h. Data indicate mean values and standard deviation (SD) of biological repeats performed in duplicate.

| **A2780** | TS protein level (%) |
| --- | --- |
| **DMSO -CHX 0h** | 100 |
| **DMSO-CHX 1h** | 77±14 |
| **DMSO-CHX 2h** | 69±20 |
| **DMSO-CHX 3h** | 45±12 |
| **E7-CHX 0h** | 18±1 |
| **E7-CHX 1h** | 17±1 |
| **E7-CHX 2h** | 10±3 |
| **E7-CHX 3h** | 8±5 |

**Figure 7G-source data 1.** Half-life calculation of exogenous hTS protein level (anti-FLAG stain) in HCT116 cells transfected with TS-Myc-DDK (0, 6 and 10h CHX) or F59A mutant (0, 6 and 10h CHX) tagged vector. Data indicate mean values and standard deviation (SD) of biological repeats performed in duplicate.

| **HCT116** | TS protein level (%) |
| --- | --- |
| **TS-MYC-DDK WT 0h** | 100 |
| **TS-MYC-DDK WT 6h** | 59±35 |
| **TS-MYC-DDK WT 10h** | 57±35 |
| **TS-MYC-DDK-F59A 0h** | 60±2 |
| **TS-MYC-DDK-F59A 6h** | 0 |
| **TS-MYC-DDK-F59A 10h** | 0 |
