# Supplementary material for: Destabilizers of the thymidylate synthase homodimer accelerate its proteasomal degradation and inhibit cancer growth
Source: eLife. 2022 Dec 7;11:e73862. doi: 10.7554/eLife.73862 (PMC9831607; doi:10.7554/eLife.73862)
Supplement: Figure 7—figure supplement 1—source data 1. [file elife-73862-fig7-figsupp1-data1.docx]

**Figure 7-figure supplement 1a,b-source data 1.**

A) Quantification of Annexin V-positive cells (% ± SD) performed using Annexin V/PI kit by flow cytometric analysis of apoptosis of A2780 and A2780/CP cells treated with cisplatin (**cDDP**) (13 µM, 72h). Data indicate mean values and standard deviation (SD) of biological repeats performed in duplicate.

| **A2780 cells** | (% ± SD) |
| --- | --- |
| **CTRL** | 6.5±1 |
| **cDDP** | 81±2 |
| **A7080/CP cells** |  |
| **CTRL** | 10±4 |
| **cDDP** | 50±1 |

**Figure 7-figure supplement 1a,b-source data 1.**

B) Quantification of Annexin V-positive cells (% ± SD) performed using Annexin V/PI kit by flow cytometric analysis of apoptosis of A2780 and A2780/CP cells treated with **E7** or **PMX**. Data indicate mean values and standard deviation (SD) of biological repeats performed in duplicate.

| **A2780 cells** | (% ± SD) |
| --- | --- |
| **CTRL** | 4.1±3 |
| **E7 - 20 µM - 48h** | 28.9±10 |
| **E7 - 40 µM - 48h** | 40.5±11 |
| **PMX - 5 µM - 48h** | 31.5±3 |
| **A7080/CP cells** |  |
| **CTRL** | 4.8±3 |
| **E7 - 20 µM - 48h** | 24.6±5 |
| **E7 - 40 µM - 48h** | 40.2±8 |
| **PMX - 5 µM - 48h** | 31.3±8 |
