# Supplementary material for: Destabilizers of the thymidylate synthase homodimer accelerate its proteasomal degradation and inhibit cancer growth
Source: eLife. 2022 Dec 7;11:e73862. doi: 10.7554/eLife.73862 (PMC9831607; doi:10.7554/eLife.73862)
Supplement: Figure 8—figure supplement 2—source data 1. — Quantitative PCR (qPCR) measurements of hTS transcripts in A2780 and A2780/CP cells treated with E7, E5, and 5FU. [file elife-73862-fig8-figsupp2-data1.pdf]

Figure 8-figure supplement 2-Source data 1. Quantitative PCR (qPCR) measurements of hTS transcripts in A2780 and A2780/CP cells treated with E7, E5 and 5FU. Data indicate mean values and standard deviation (SD) of biological repeats performed in duplicate.

**A2780 cells**	Relative hTS mRNA
**CTRL**	1.00
**E7 – 12h - 20 µM**	0.51±0.04
**E7 – 12h - 40 µM**	0.47±0.10
**E5 – 12h - 30 µM**	0.54±0.46
**E5 – 12h - 60 µM**	0.53±0.23
**5FU - 24h - 5µM**	0.70±0.04
**5FU - 72h - 5µM**	0.50±0.24
**A2780/CP**	
**CTRL**	1.00
**E7 – 12h - 20 µM**	0.66±0.11
**E7 – 12h - 40 µM**	0.53±0.01
**E5 – 12h - 30 µM**	0.74±0.08
**E5 – 12h - 60 µM**	0.56±0.01
**5FU - 24h - 5µM**	0.57±0.07
**5FU - 72h - 5µM**	0.29±0.09
