# Supplementary material for: Destabilizers of the thymidylate synthase homodimer accelerate its proteasomal degradation and inhibit cancer growth
Source: eLife. 2022 Dec 7;11:e73862. doi: 10.7554/eLife.73862 (PMC9831607; doi:10.7554/eLife.73862)
Supplement: Supplementary file 1. [file elife-73862-supp1.docx]

**Computational protein-pocket identification for tethering**

**Supplementary file 1A.**

**Pockets found at the dimer interface of the active hTS (PDB ID: 1HVY).** During the MD simulations, all pockets were dynamic and changed size, some disappeared or reappeared (see Table S2).

| **Pocket** | **Residues forming the pocket** | **Size (Å^3^)^a^** | **RNA binding residues^b^** | **Other properties** |
| --- | --- | --- | --- | --- |
| ***Pockets found in the crystal structure (monomer, A chain)*** | | | | |
| Y202 | R64, Q200, F201^c^, Y202, S209, C210^c^, Q211, D247, I249 | 22 | No | Not found in the dimeric structure since F59' of the other monomer fills the pocket. (Fig. S1). Interactions with polar, hydrophobic and aromatic residues are possible. |
| K47a | K47, D49, R50, T51, T55, S57, Y258, D254, H256, S216, G217 | n.d.^d^ | Yes: K47, D254^e^ | This pocket is part of the active site (Fig. S1). In a dimer, it is filled with R175' from the other monomer. Interactions with polar, hydrophobic and aromatic residues are possible. There are several energetically favorable water binding sites in front of the active site (PDB ID: 1HVY). |
| K47b | K47, S57, F59, D254 | 19.4 | Yes: K47, F59, D254^e^ | This pocket is at the left side of the active site front (Fig. S1). It is not found in the dimer because it is filled with Y202' of the other monomer. Interactions with polar, aromatic and especially hydrophobic residues are possible. |
| I178 | Q160, R163, V164, T167, R176, I177, I178, M179 | 32.7 | Yes: R176 | This pocket can also be found in the dimer. Interactions with polar, hydrophobic and aromatic residues are possible. Especially water probe was favored, consistent with the crystal water found in the site. |
| Y213 | A197, L198, Q211, Y213 | 3.9 | Yes: A197, Y213^f^ | In the dimer this pocket is partially filled with the Y213' from the other monomer. It is a very hydrophobic pocket. |
| ***Pockets that appeared during the MD simulations (monomer, A chain)*** | | | | |
| W182 | F137, Q138, F142, G143, A144, Q160, C180, W182, N183, R185, D186^g^ | 101.4^g^ | Yes: F142, R185 | W182 is within the active site loop (residues 181-197) that twists around 180° when hTS is activated/inactivated. Residue F142 divides the pocket into two compartments. A narrow region for favorable polar or aromatic interactions is found in the middle of the pocket and a large region for hydrophobic interaction at the mouth of the pocket. |
| C195 | W182, P194, C195, H196, A197, R215^h^ | 59.6^h^ | Yes: C195, A197, R215 | C195 is the catalytic residue and resides on the other side of the active site loop than W182. |
| ***Pockets found in the crystal structure (dimer)*** | | | | |
| A^i^ | V158, Q160, R163, V164, T167, D174, R176, I177, I178, W182’ ^j^, P184’, L187’, P188’, P193’ | 232/362 | Yes: V158, R176, P184 | These pockets reside between the active site loop and the ‘R175 bump’ (residues 170-175) in the dimeric form of hTS and the I178 pocket of the monomeric hTS is part of it. |
| B^i^ | H39, C43, G44, V45, S57, V58, F59, R64’, R68’, Y202’, V204’, E207’, S209’, D247’, I249’ | 38/210 | Yes: H39, C43, G44, V45, V58, F59 | These pockets reside between the beta sheet kink and the ‘C20 peptide’^k^ (residues 198-217). The region below the main Y202 pocket is part of these pockets. |
| C | F142, F142’, C180, W182, W182’, P184 | 25 | Yes: F142, P184 | This symmetric pocket resides at the crossroads of the interface loops (residues 141-159) and active site loops (i.e., between the A pockets). In the inactive hTS, this pocket is deeper and larger than in the active hTS due to the conformational change of the active site loops upon inactivation. |
| D^i^ | K47, D48, D49, T55, S57, F59, D254, H256, P172’, D173’, D174’,R175’, I177’, Q200’, Y202’, F201’, V203’ | 143/149 | Yes: K47, F59, D254^e^ | These are groups of small pockets in front of the dUMP phosphate binding site. They line the K47 pockets of the monomeric hTS and are favorable binding sites for water. |

^a^Size as calculated by CASTp;

^b^RNA binding residues as predicted with the KYG server (both active and inactive hTS conformations);

^c^Backbone atoms only;

^d^Not possible to determine the size as the pocket is part of the active site cavity;

^e^In the inactive conformation (PDB ID: 1YPV);

^f^Only in the active conformation;

^g^At 7 ns of the explicit water MD simulation (with crystal water included);

^h^At 4 ns of the explicit water MD simulation (with crystal water included);

^i^Two similar pockets found due to symmetry.

^j^(’) after a residue name refers to the residue in the opposite monomer;

^k^A 20-mer peptide at the hTS dimer interface (*21*).

**Supplementary file 1B**

**Interface pockets at the 1HVY monomer during the explicit water simulation with crystal water molecules included (without dUMP).** Pockets are named according to the data in Table S1.

| **Time**  **(ns)** | **Size of a pocket (Å^3^) determined by CASTp^a^** | | | | | |
| --- | --- | --- | --- | --- | --- | --- |
|  | **Y202 pocket (& around)^b^** | **I178 pocket** | **Y213 pocket** | **K47b pocket^c^** | **W182 pocket** | **C195 pocket** |
| 0 | 169.4 | 32.7 | 3.9 | 19.4 | - | - |
| 1 | 37.1 | 237 | - | - | 22.8 | 14.5 |
| 2 | 271.6 | 11.6 | 46.3 | 24 | 11.8 | 24.6 |
| 3 | 392.5 | 42.7 | 59.6 | - | 14.5 | 19.8 |
| 4 | 510.6 | 63.1 | 53.1 | - | 17.4 | 59.6 |
| 5 | 276.3 | 42.2 | - | 93.1 | - | 34 |
| 6 | 584.3 | 14.8 | - | - | - | - |
| 7 | 101.5 | - | - | - | 101.4 | - |

^a^Sizes of the pockets depend on the method CASTp uses for their calculation. In principle, according to CASTp, a phenyl ring can fit into a pocket of ca. 20 Å^3^, as e.g. for the K47b pocket in the crystal structure).

^b^ The region surrounding the Y202 pocket is included here; thus the volumes are much larger than the pocket itself (Table S1). Y202 can block the main pocket by moving its side chain (in the monomer simulations).

^c^The K47a pocket is always part of the large active site cavity, so its size cannot be followed separately. (see Fig. S1).
