# Supplementary material for: Destabilizers of the thymidylate synthase homodimer accelerate its proteasomal degradation and inhibit cancer growth
Source: eLife. 2022 Dec 7;11:e73862. doi: 10.7554/eLife.73862 (PMC9831607; doi:10.7554/eLife.73862)
Supplement: Supplementary file 2. [file elife-73862-supp2.docx]

**X-ray crystallography**

**Supplementary file 2A**

**Data collection and refinement statistics** (data in parenthesis refer to the highest resolution shell).

| Protein | **hTS mutant Y202C** | **hTS mutant C195S-Y202C** |
| --- | --- | --- |
| PDB code | **4O1U** | **4O1X** |
| **DATA COLLECTION STATISTICS** | | |
| Beamline, λ(Å) | ESRF ID29, 0.9360 | ESRF ID23-2, 0.873 |
| Space group | P3_1_ | P2_1_2_1_2_1_ |
| Δφ (°) | 1 | 0.5 |
| Cell dimensions (Å, °)  a  b  c | 96.23  96.23  83.77 | 94.93  95.52  131.59 |
| Resolution (Å) | 48.12 - 2.26 (2.38 – 2.26) | 38.65 – 2.32 (2.45 – 2.32) |
| N mol/asym. unit | 2 (1 dimer) | 4 (2 dimers) |
| Observed reflections | 151047 (22511) | 212942 (30276) |
| Unique reflections | 39688 (5882) | 51794 (7464) |
| Completeness (%) | 97.6 (98.6) | 99.0 (99.0) |
| Rmerge (%) | 6.7 (37.8) | 8.5 (36.6) |
| I/σ(I) | 12.0 (3.7) | 10.4 (4.0) |
| Multiplicity | 3.8 (3.8) | 4.1 (4.1) |
| **REFINEMENT STATISTICS** | | |
| Resolution range (Å) | 48.12 – 2.26 (2.32 – 2.26) | 38.49 – 2.32 (2.38 – 2.32) |
| R_cryst_ (%) | 17.2 (22.7) | 20.6 (27.5) |
| n. of ref. in R_free set_, R_free_ (%) | 1972, 21.0 (27.7) | 2628, 26.0 (39.4) |
| Total atoms  protein  waters  sulfate ions  other (ethylene glycol, chloride) | 4475  4240  186  30  19 | 9482  9169  250  40  23 |
| Mean B value (Å^2^) | 54.17 | 32.89 |
| RMSD bond lengths (Å) | 0.010 | 0.011 |
| RMSD bond angles (°) | 1.435 | 1.532 |
| RMSD chiral volumes | 0.099 | 0.097 |
| RMSD planes (Å) | 0.006 | 0.007 |
| Est. error on coord. based on  R value (Å) | 0.19 | 0.45 |
| Ramachandran plot (%)  Favored  Allowed  Not allowed | 90.8  9.2  0.0 | 89.6  10.4  0.0 |

**Supplementary file 2B.**

**Solvent accessibility of the cysteine residues in hTS mutants and their chemical modifications upon reaction with β-mercaptoethanol.** SCH = S-methyl-thio-cysteine; CME = S,S-(2-hydroxyethyl) thiocysteine.

| Decreasing solvent accessibility | Solvent accessibility | Cysteine residue number | Chemical Modification | |
| --- | --- | --- | --- | --- |
|  |  |  | Y202C | C195S_Y202C |
|  | Fully accessible | 43 | SCH | CME |
|  | Partially accessible | 180 | SCH | Cys (possibly modified: weak electron density beyond S) |
|  | Partially accessible | 195 | SCH | Ser |
|  | Partially accessible | 202 | CME | CME |
|  | Almost buried | 199 | CME | CME |
|  | Completely buried | 210 | Cys | Cys |
