# Supplementary material for: Destabilizers of the thymidylate synthase homodimer accelerate its proteasomal degradation and inhibit cancer growth
Source: eLife. 2022 Dec 7;11:e73862. doi: 10.7554/eLife.73862 (PMC9831607; doi:10.7554/eLife.73862)
Supplement: Supplementary file 3. [file elife-73862-supp3.docx]

**Mass spectrometry data.**

**Supplementary file 3A**

**Disulfide library screened by mass spectrometry.**

| Compound | Properties | Commercial Codes | Structures |
| --- | --- | --- | --- |
| A1 | MW: 274.358  Log P: 3.110  N Heavy Atoms: 9+9 | NCI: NSC209  ZINC: 1555356 |  |
| A2 | MW: 435.39  Log P:4.964  N Heavy Atoms: 13+13 | NCI: NSC659162  ZINC: 1636936 |  |
| A3 | MW: 180.248  Log P: -1.014  N Heavy Atoms: 5+5 | NCI: NSC28727  ZINC: 1646639 |  |
| A4 | MW: 154.251  Log P: 0.380  N Heavy Atoms: 4+4 | NCI: NSC33920  ZINC: 1665970 |  |
| A5 | MW: 312.325  Log P: 2.780  N Heavy Atoms: 10+10 | NCI: NSC149336  ZINC: 1734461 |  |
| A6 | MW: 240.300  Log P: -5.196  N Heavy Atoms: 7+7 | NCI: NSC13203  ZINC: 1529198 |  |
| A7 | MW: 268.354  Log P: -4.214  N Heavy Atoms: 8+8 | NCI: NSC11337  ZINC: 1529294 |  |
| A8 | MW: 332.394  Log P: 2.768  N Heavy Atoms: 11+11 | NCI: NSC211  ZINC: 1555358 |  |
| A9 ^a^ | MW: 296.474  Log P: 3.992  N Heavy Atoms: 8+10 | NCI: NSC261199  ZINC: 1558189 |  |
| A10 | MW: 534.694  Log P: 4.136  N Heavy Atoms: 17+17 | NCI: NSC273905  ZINC: 1561354 |  |
| A11 | MW: 240.300  Log P: -5.694  N Heavy Atoms: 7+7 | NCI: NSC302851  ZINC: 1567219 |  |
| A12 ^a^ | MW: 246.369  Log P: 1.442  N Heavy Atoms: 4+11 | NCI: NSC3420025  ZINC: 1579481 |  |
| A13 ^a^ | MW: 213.296  Log P: 3.333  N Heavy Atoms: 3+10 | NCI: NSC342013  ZINC: 1579486 |  |
| A14 ^a^ | MW: 200.344  Log P: 2.318  N Heavy Atoms: 4+8 | NCI: NSC342020  ZINC: 1579492 |  |
| A15 ^a^ | MW: 272.321  Log P: -0.643  N Heavy Atoms: 7+10 | NCI: NSC342023  ZINC: 1579494 |  |
| A16 | MW: 380.53  Log P: 4.388  N Heavy Atoms: 13+13 | NCI: NSC3286  ZINC: 1666690 |  |
| A17 | MW: 300.402  Log P: 4.188  N Heavy Atoms: 10+10 | NCI: NSC35825  ZINC: 1668414 |  |
| A18 | MW: 334.37  Log P: 2.584  N Heavy Atoms: 11+11 | NCI: NSC38069  ZINC: 1670426 |  |
| A19 | MW: 433.329  Log P: 3.812  N Heavy Atoms: 13+13 | NCI: NSC39633  ZINC: 1671480 |  |
| A20 ^a^ | MW: 373.424  Log P: -0.922  N Heavy Atoms: 7+17 | NCI: NSC43135  ZINC: 1675892 |  |
| A21 | MW: 424.458  Log P: 0.462  N Heavy Atoms: 14+14 | NCI: NSC71895  ZINC: 1697318 |  |
| A22 | MW: 416.467  Log P: 4.628  N Heavy Atoms: 14+14 | NCI: NSC108934  ZINC: 1701075 |  |
| A23 | MW: 508.617  Log P: 1.992  N Heavy Atoms: 17+17 | NCI: NSC110311  ZINC: 1702746 |  |
| A24 | MW: 304.341  Log P: 3.668  N Heavy Atoms: 10+10 | NCI: NSC110311  ZINC: 1704344 |  |
| A25 | MW: 353.438  Log P: 4.974  N Heavy Atoms: 12+12 | NCI: NSC125034  ZINC: 1714235 |  |
| A26 | MW: 420.592  Log P: 4.408  N Heavy Atoms: 13+13 | NCI: NSC125828  ZINC: 1714842 |  |
| A27 | MW: 352.427  Log P: 2.342  N Heavy Atoms: 11+11 | NCI: NSC175829  ZINC: 1716349 |  |
| A28 | MW: 406.431  Log P: 2.400  N Heavy Atoms: 13+13 | NCI: NSC175830  ZINC: 1716350 |  |
| A29 ^a^ | MW: 181.276  Log P: -1.22  N Heavy Atoms: 3+7 | NCI: NSC135126  ZINC: 1721731 |  |
| A30 | MW: 304.341  Log P: 3.64  N Heavy Atoms: 10+10 | NCI: NSC210281  ZINC: 1745667 |  |
| A31 ^a^ | MW: 383.425  Log P: 4.847  N Heavy Atoms: 8+16 | NCI: NSC213718  ZINC: 1752388 |  |
| A32 | MW: 268.486  Log P: 0.502  N Heavy Atoms: 7+7 | NCI: NSC18421  ZINC: 1769219 |  |
| A33 ^a^ | MW: 244.354  Log P: 1.979  N Heavy Atoms: 4+11 | NCI: NSC342027  ZINC: 1579497 |  |
| A34 | MW: 346.426  Log P: -0.122  N Heavy Atoms: 11+11 | NCI: NSC350993  ZINC: 1581670 |  |
| A35 | MW: 460.654  Log P: 4.418  N Heavy Atoms: 14+14 | NCI: NSC363956  ZINC: 1585472 |  |
| A36 ^a^ | MW: 248.342  Log P: 2.330  N Heavy Atoms: 4+11 | NCI: NSC369423  ZINC: 1587145 |  |
| A37 | MW: 354.400  Log P: 3.734  N Heavy Atoms: 12+12 | NCI: NSC370926  ZINC: 1587470 |  |
| A38 | MW: 364.393  Log P: 4.058  N Heavy Atoms: 12+12 | NCI: NSC372683  ZINC: 1588809 |  |
| A39 | MW: 354.535  Log P: -0.49  N Heavy Atoms: 10+10 | NCI: NSC403854  ZINC: 1596138 |  |
| A40 | MW: 462.587  Log P: 3.728  N Heavy Atoms: 16+16 | NCI: NSC611506  ZINC: 1611876 |  |
| A41 ^a^ | MW: 197.299  Log P: -3.390  N Heavy Atoms: 4+7 | NCI: NSC161601  ZINC: 1623380 |  |
| A42 | MW: 210.271  Log P: 1.608  N Heavy Atoms: 6+6 | NCI: NSC638710  ZINC: 1625711 |  |
| A43 ^a^ | MW: 178.272  Log P: 2.648  N Heavy Atoms: 4+6 | NCI: NSC96660  ZINC: 1626922 |  |
| A44 ^a^ | MW: 303.292  Log P: 2.194  N Heavy Atoms: 6+13 | NCI: NSC96669  ZINC: 1626930 |  |
| A45 ^a^ | MW: 362.443  Log P: 4.087  N Heavy Atoms: 10+14 | NCI: NSC96696  ZINC: 1626956 |  |
| A46 | MW: 404.548  Log P: 5.792  N Heavy Atoms: 12+12 | NCI: NSC97416  ZINC: 1632630 |  |
| A47 | MW: 289.203  Log P: 4.406  N Heavy Atoms: 8+8 | NCI: NSC98831  ZINC: 1648618 |  |
| A48 | MW: 238.324  Log P: 1.828  N Heavy Atoms: 7+7 | NCI: NSC308782  ZINC: 1568750 |  |
| A49 | MW: 460.654  Log P: 4.346  N Heavy Atoms: 14+14 | NCI: NSC342021  ZINC: 1579493 |  |
| A50 ^a^ | MW: 270.412  Log P: -2.552  N Heavy Atoms: 7+8 | NCI: NSC342029  ZINC: 1579499 |  |
| A51 | MW: 442.551  Log P: 2.012  N Heavy Atoms: 15+15 | NCI: NSC405812  ZINC: 1598776 |  |
| A52 | MW: 272.432  Log P: -0.834  N Heavy Atoms: 7+7 | NCI: NSC14558  ZINC: 1653104 |  |
| A53 | MW: 254.289  Log P: -1.58  N Heavy Atoms: 8+8 | NCI: NSC106685  ZINC: 1697982 |  |
| A54 | MW: 340.415  Log P: -2.662  N Heavy Atoms: 8+8 | NCI: NSC126672  ZINC: 1715307 |  |
| A55 | MW: 262.346  Log P: 4.702  N Heavy Atoms: 8+8 | NCI: NSC15869  ZINC: 1733696 |  |
| TCP | MW: 654.884  Log P: 4.088  N Heavy Atoms: 21+21 |  |  |

^a^ For asymmetric disulphide compounds the two possible binding fragments are indicated by “a” and “b”.

**Supplementary file 3B**

**Mass spectrometry screening results: compounds detected bound to hTS C195S-Y202C.**

| Compound | MALDI | ESI-QToF |
| --- | --- | --- |
| **A2** | detected | not detected |
| **A4** | detected | detected |
| **A5** | detected | detected |
| **A6** | detected | detected |
| **A7** | detected | not detected |
| **A9** | detected | not detected |
| **A10** | detected | detected |
| **A11** | detected | not detected |
| **A14** | detected (both fragments) | detected (both fragments) |
| **A15** | detected (both fragments) | detected (both fragments) |
| **A16** | not detected | detected |
| **A20** | detected (fragment b) | detected (fragment b) |
| **A21** | detected | not detected |
| **A22** | detected | not detected |
| **A25** | detected | not detected |
| **A26** | detected | not detected |
| **A27** | detected | not detected |
| **A28** | detected | not detected |
| **A29** | detected (both fragments) | detected (both fragments) |
| **A30** | detected | detected |
| **A36** | detected (both fragments) | detected (fragment b) |
| **A37** | detected | not detected |
| **A38** | detected | detected |
| **A41** | detected | not detected |
| **A42** | detected | not detected |
| **A43** | detected | detected (both fragments) |
| **A47** | detected | detected |
| **A49** | detected | not detected |
| **A55** | detected | not detected |
| **TCP** | detected | detected |

**Supplementary file 3C**

**Results of the mass spectrometry experiments on C195S-Y202C hTS protein-ligand complexes digested with trypsin and analyzed by MALDI (M) and ESI (E) MS**. If the ligand is not identified through E it is indicated as (E-).

| Compound | CYS 180 | | | | CYS 199, 202, 210 | |
| --- | --- | --- | --- | --- | --- | --- |
|  | (177)- (185)  IIM**C**AWNPR  (sequence A) | | (176) –(185)  RIIM**C**AWNPR  (sequence B) | | (186)- (215)  DLPLMALPPSHAL**C**QF**C**VVNSELS**C**QLYQR  (sequence C) | |
|  | Cam (1x)  1160.5708 Da | Lig (1x)^a^ | Cam (1x)  1316.6720 Da | Lig (1x)^a^ | Cam (3x)  3546.7006 Da | Lig (1x)  Cam (2x)^a^ |
| **A5** | M E | M E | ND^b^ | NDb | M E | M |
| **A6** | M E | ND^b^ | ND^b^ | M E | M E- | ND^b^ |
| **A10** | M E | M | E | ND^b^ | M E | M |
| **A15 (both fragments)** | M E | E(a) | ND^b^ | M(a)-E(ab) | M E- | ND^b^ |
| **A20 (fragment b)** | M E | M(b) E(ab) | E | NDb | M E- | M(b) E(b) |
| **A38** | M E | ND^b^ | ND^b^ | M E | M E | ND^b^ |
| **TCP** | M E | M E | ND^b^ | ND^b^ | M E | M |

^a^The ligands bound to the Cys-containing peptides were identified by replacing the MW of the carbamidomethylic group (56 Da) with the MW of the thiol originated from the disulphides on the prototypic reporter ions 1160.5, 1316.7, 3546.7. For symmetric disulphides, the MW of the tethered-ligand corresponds to half of the MW of the molecule; for asymmetric disulphides, the MW of the tethered-ligand corresponds to the MW of either the **a** or **b** portions of the molecule; ^b^ ND= not detected
