# Supplementary material for: Destabilizers of the thymidylate synthase homodimer accelerate its proteasomal degradation and inhibit cancer growth
Source: eLife. 2022 Dec 7;11:e73862. doi: 10.7554/eLife.73862 (PMC9831607; doi:10.7554/eLife.73862)
Supplement: Supplementary file 4. [file elife-73862-supp4.docx]

Supplementary file 4A

**Compounds B1-B26: structures, FRET and kinetic inhibition data against hTS. ΔΦ_fret_ = change in FRET efficiency with respect to untreated hTS.**

| Compound | Formula | % enzyme activity inhibition^a^ | ***ΔΦ_fret_^b^*** |
| --- | --- | --- | --- |
| **B1** |  | 20 | 0 |
| **B2** |  | 20 | 0 |
| **B3** |  | 15 | 0 |
| **B4** |  | ND^c^ | 0 |
| **B5** |  | ND | 0 |
| **B6** |  | ND | 0 |
| **B7** |  | ND | 0 |
| **B8** |  | 30 | 0 |
| **B9** |  | 40 | 0 |
| **B10** |  | 15 | 0 |
| **B11** |  | 85 | 0 |
| **B12** |  | 15 | -0.13 |
| **B13** |  | 25 | 0 |
| **B14** |  | 25 | 0 |
| **B15** |  | 45 | 0 |
| **B16** |  | ND | 0 |
| **B17** |  | 35 | 0 |
| **B18** |  | 35 | 0 |
| **B19** |  | 15 | 0 |
| **B20** |  | 40 | 0 |
| **B21** |  | 15 | 0 |
| **B22** |  | 40 | 0 |
| **B23** |  | ND | 0 |
| **B24** |  | 30 | 0 |
| **B25** |  | 15 | 0 |
| **B26** |  | ND | -0.02 |

^a^ % inhibition was measured at 50 μM conc., unless otherwise stated; standard error (SE) +/-20% of the displayed value.

^b^ measured at 50 μM; the value is the result of the subtraction of the ΔΦ_fret_ value obtained in the presence of DMSO alone (-0.1) from that obtained in the presence of the tested compounds; standard error (SE) +/-15% of the displayed value;

^c^ ND not active at the concentrations tested.

Supplementary file 4B

**Compounds C1-C11: structures, FRET and kinetic inhibition data against hTS.**

| **Compound** |  | **Synthetic Scheme** | ***IC_50_ (μM)^b^*** | ***ΔΦ _fret_^b^*** |
| --- | --- | --- | --- | --- |
| **C1** |  | 2 | ND^c^ | -0.31 |
| **C2** |  | 1 | 83 | -0.37 |
| **C3** |  | 1 | 5.25 | -0.5 |
| **C4** |  | 1 | 246 | -0.30 |
| **C5** |  | 1 | 76 | -0.33 |
| **C6** |  | 3 | ND | -0.09 |
| **C7** |  | 3 | ND | -0.10 |
| **C8** |  | 3 | 30 | -067^d^ |
| **C9** |  | 3 | ND | -0.05 |
| **C10** |  | 2 | ND | -0.56 |
| **C11** |  | 3 | 75 | -0.21 |

^a^ IC_50_ data unless otherwise stated show a standard error (SE) +/-20% of the displayed value.

^b^ measured at 50 μM; the value is the result of the subtraction of the ΔΦ_fret_ value obtained in the presence of DMSO alone (-0.1) from that obtained in the presence of the tested compounds; standard error (SE) +/-15% of the displayed value;

^c^ ND not active at 50 μM ;

^d^ measured at 100 μM.

Supplementary file 4C

**Compounds D1-D14: structures, FRET and kinetic inhibition data against hTS.**

| **Compound** | R | **Synthetic Scheme** | ***IC_50_ (μM)^a^*** | ***ΔΦ_fret_^b^*** |
| --- | --- | --- | --- | --- |
| **D1** |  | 4 | ND^c^ | 0 |
| **D2** |  | 4 | ND | 0 |
| **D3** |  | 4 | ND | 0 |
| **D4** |  | 4 | ND | -0.09 |
| **D5** |  | 1 | 40 | -0.24 |
| **D6** |  | 1 | 53 | -0.22 |
| **D7** |  | 1 | 42 | -0.33 |
| **D8** |  | 1 | 47 | -0.14 |
| **D9** |  | 2 | 60 | -0.36^d^ |
| **D10** |  | 2 | 85 | -0.26 |
| **D11** |  | 1 | ND | 0 |
| **D12** |  | 1 | 105 | -0.22 |
| **D13** |  | 1 | ND | 0 |
| **D14** |  | 1 | ND | 0 |

^a^ IC_50_ data unless otherwise stated show a standard error (SE) +/-20% of the displayed value.

^b^ measured at 50 μM; the value is the result of the subtraction of the ΔΦ_fret_ value obtained in the presence of DMSO alone (-0.1) from that obtained in the presence of the tested compounds; standard error (SE) +/-15% of the displayed value;

^c^ ND not active at 50 μM ;

^d^ measured at 100 μM.

Supplementary file 4D

**Compounds E1-E7: structures, FRET and kinetic inhibition data against hTS.**

| **Compound** |  | **Synthetic Scheme** | ***IC_50_ (μM)^a^*** | ***ΔΦ_fret_^b^*** |
| --- | --- | --- | --- | --- |
| **E1** |  | 3 | 44 | -0.32 |
| **E2** |  | 3 | ND^c^ | 0 |
| **E3** |  | 3 | 23 | -0.56 |
| **E4** |  | 3 | 40 | -0.87^d^ |
| **E5** |  | 3 | 40 | -0.24 |
| **E6** |  | 3 | 10 | -0.24 |
| **E7** |  | 3 | 7^e^ | -0.1 at 10 µM^f^ |

^a^ IC_50_ data unless otherwise stated show a standard error (SE) +/-20% of the displayed value.

^b^measured at 50 μM; the value is the result of the subtraction of the ΔΦ_fret_ value obtained in the presence of DMSO alone (-0.1) from that obtained in the presence of the tested compounds; standard error (SE) +/-15% of the displayed value; all experiments were repeated 3 times.

^c^ ND not active at 50 µM;

^d^ measured at 100 μM;

^e^ IC_50_ apparent value, due to low solubility;

^f^ΔΦ_fret_ only detectable at 10μM, due to low solubility.

Supplementary file 4E

**Compounds F1-F4: structures, FRET and kinetic inhibition data against hTS.**

|  | | | | | |
| --- | --- | --- | --- | --- | --- |
| Compound | R_1_ | R_2_ | **Scheme** | ***IC_50_ (μM)*** | ***ΔΦ_fret_^a^*** |
| **F1** | -H | -H | 5 | ND^b^ | -0.02 |
| **F2** | -OCH_3_ | -OCH_3_ | 6 | ND | -0.35 |
| **F3** | -COOCH_3_ | -H | 6 | ND | -0.13 |
| **F4** | -COOH | -H | 6 | ND | -0.04 |

^a^ measured at 50 μM; the value is the result of the subtraction of the ΔΦ_fret_ value obtained in the presence of DMSO alone (-0.1) from that obtained in the presence of the tested compounds; standard error (SE) +/-15% of the displayed value; all experiments were repeated at least 3 times.

^b^ ND not active at 50 µM.
