# Supplementary material for: Destabilizers of the thymidylate synthase homodimer accelerate its proteasomal degradation and inhibit cancer growth
Source: eLife. 2022 Dec 7;11:e73862. doi: 10.7554/eLife.73862 (PMC9831607; doi:10.7554/eLife.73862)
Supplement: Supplementary file 5. [file elife-73862-supp5.docx]

**UHPLC-HRMS quantitation of the dimer disrupters in AEX experiments**

The amount of dimer disrupter under each peak eluted from FPLC (2mL per fraction) was measured with LC-MS (UltiMate 3000 UHPLC tandem Orbitrap Q-Ex, Centro interdipartimentale grandi strumenti, CIGS, UniMORE) after appropriate sample preparation.

100uL of each fraction were pipetted in a 1.5uL Eppendorf tube and diluted to 500uL with Acetonitrile with 0.5% Formic Acid (FA MS grade, Lichropur) to crash proteins. Samples were incubated for 2h at room temperature and centrifuged at 14.000 g for 20min to pelletize the precipitated materials. 40uL of supernatant were put in conical vials for the instrument autosampler.

Calibration solutions for regression curve were prepared in protein buffer as described above, without hTS enzyme, in a concentration range from 1nM to 500nM. Calculated R^2^ was 0.9976 for **C3** curve and 0.9971 for **E5** curve (**Figure 1**).

**C3** and **E7** were separated with an Hypersil GOLD 100x2.1mm, 1.9mm pore size column (Thermo Fisher Scientific), termostatted at 30C, with 0.1% FA in water (A) and Acetontrile with 0.1% FA (B).

LC gradient was set as in **Table 1.** MS parameters were set as follows. Scan mode: ESI-, MS resolution: 140.000, acquisition mode: tSIM with LC1130 and LC1296 exact masses inclusion list active (monoisotopic masses calculated with FreeStyle, Thermo Fisher), acquisition window: 0.5 m/z; max it 287ms; AGC 3x10^6^. An ESI negative source calibration was performed with a commercial low-mid masses standard mix from Thermo Fisher the same day of the analysis to ensure reproducibility. **Figure 2** reports the exact monoisotopic masses for the two compounds. Each measurement was performed in replicate. Genesis quantification Algorithm on FreeStyle software was applied to all plots with the following calculation parameters: Percent of interest peak: 10, minimum % S/N: 10; S/N threshold: 10; automatic baseline (Base Peak Range).

The exact results of the quantitations were used to generate Figure 3-figure supplement 2.

**Figure 1**. Calibration curves for LC C3 (left) and E7 (right)


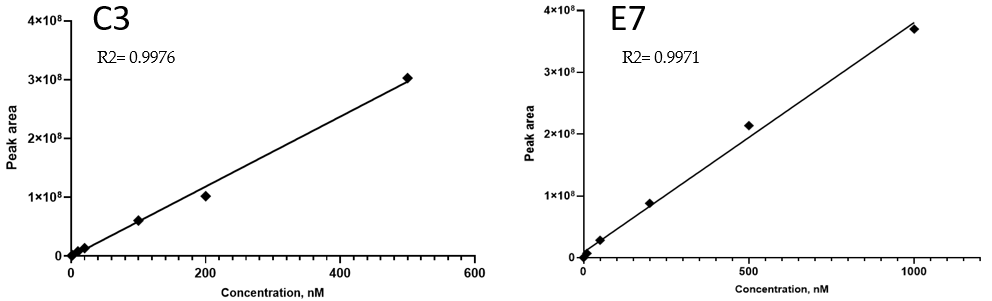


**Table 1.** UHPLC gradient employed for C3 and E7 quantitation.

| Time | %B |
| --- | --- |
| 0 | 5 |
| 10 | 60 |
| 11 | 98 |
| 15 | 98 |
| 20 | 5 |


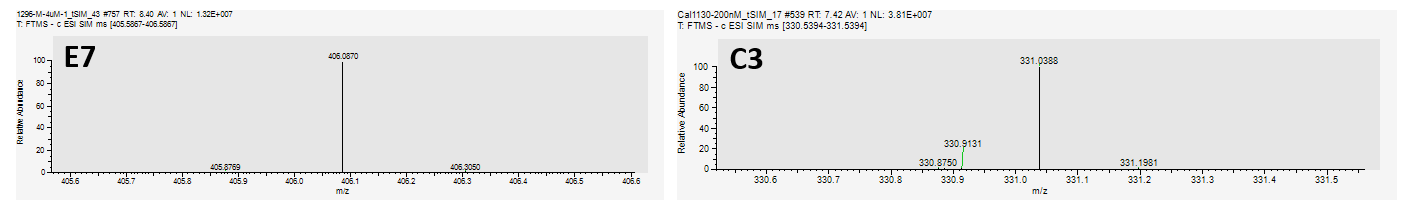


**Figure 2.** HRMS of E7 (left), 406.0870 m/z [M-H]-, and C3 (right), 331.0388 m/z [M-H]-, acquired in tSIM mode, 0.5m/z isolation window.
