# Supplementary material for: Destabilizers of the thymidylate synthase homodimer accelerate its proteasomal degradation and inhibit cancer growth
Source: eLife. 2022 Dec 7;11:e73862. doi: 10.7554/eLife.73862 (PMC9831607; doi:10.7554/eLife.73862)
Supplement: Supplementary file 6. [file elife-73862-supp6.docx]

**Supplementary file 6. Cancer cell growth inhibition and pharmacokinetic.**

**A. Cancer growth inhibition by compounds E5, E7**. IC_50_ values (µM ± SE) for compounds **E5**, **E7**, obtained after 72 h treatment of PDAC-2, PDAC-5 and LPc167 pancreatic cancer cells, HT29, HCT116 and LoVo human colorectal cancer cells Data indicate mean values and standard deviation from at least two experiments performed in duplicate. P values were calculated with two-sided Student’s *t*-test and ANOVA followed by the Tukey's multiple comparison. * *p* < 0.05; ** *p* <0.01; *** *p* < 0.001 (n=3).

| **Compounds** | **PDAC-2** | **PDAC-5** | **LPc167** | **HT29 cells** | **HCT116 cells** | **LoVo cells** |
| --- | --- | --- | --- | --- | --- | --- |
| **5-FU** | 3.00 ± 0.7 | 4.37 ± 2.1 | > 100 | 14.2 ± 3.0 | 13.5 ± 1.5 | 7.9 ± 1.2 |
| **E5** | 32.5 ± 6.5 | 17.9 ± 1.0 | 10.61±1.25 | 70.5 ± 1.6 | 61.8 ± 1.8 | 52.1 ± 9.7 |
| **E7** | 9.1 ± 0.4 | 11.6 ± 1.6 | 11.32±2.37 | 34.5 ± 3.0 | 35.5 ± 0.3 | 23.3 ± 9.0 |

**B. Cancer growth inhibition by hTS dimer disrupters.** IC_50_ values (µM ± SE) for selected compounds obtained after 72 h treatment of 2008, C13*, A2780, A2780/CP, IGROV1 and TOV112D human ovarian cancer cells. Data indicate mean values and standard deviation from at least two experiments performed in duplicate. P values were calculated with two-sided Student’s *t*-test and ANOVA followed by the Tukey's multiple comparison. * *p* < 0.05; ** *p* <0.01; *** *p* < 0.001 (n=3-5)

| **Compounds** | **2008**  **cells** | **C13***  **cells** | **A2780 cells** | **A2780/CP cells** | **IGROV1 cells** | **TOV112D cells** |
| --- | --- | --- | --- | --- | --- | --- |
| **5-FU** | 6.4±0.7 | 9.3±1 | 8.5±0.8 | 11.6±1.2 | 16.9±1 | ND |
| **C2** | >100 | >100 | >100 | >100 | >100 | >100 |
| **C3** | >100 | >100 | >100 | >100 | >100 | 99.1±11 |
| **C4** | >100 | >100 | >100 | >100 | >100 | >100 |
| **C5** | >100 | >100 | >100 | >100 | >100 | >100 |
| **C8** | 98±7 | >100 | >100 | >100 | >100 | >100 |
| **C11** | >100 | >100 | >100 | >100 | >100 | >100 |
| **D5** | >100 | >100 | >100 | >100 | >100 | >100 |
| **D6** | >100 | >100 | >100 | >100 | >100 | >100 |
| **D7** | >100 | >100 | >100 | >100 | >100 | >100 |
| **D8** | >100 | >100 | >100 | >100 | >100 | >100 |
| **D9** | >100 | >100 | >100 | >100 | >100 | >100 |
| **D12** | >100 | >100 | >100 | >100 | >100 | >100 |
| **E1** | 77±4 | >100 | >100 | >100 | >100 | >100 |
| **E3** | >100 | >100 | 76.6±9 | >100 | >100 | 72.2±9 |
| **E4** | 55.8±7 | 76.3±9 | 88.3±11 | >100 | >100 | 88.5±9 |
| **E5** | 29.8±4 | 38.3±5 | 28.8±4 | 34.3±5 | 63.9±8 | 60.5±7 |
| **E6** | 61±6 | 50.5±2 | 19.5±2 | 18.7±1 | 39.1±3 | 41.5±7 |
| **E7** | 14.6±1 | 21.1±2 | 10.1±0.3 | 21.5±3 | 37.4±6 | 31.±2 |

**C. PK parameters for E5 and E7 after single intravenous (IV) or oral administration (PO).** Cmax (maximum plasma concentration); Tmax (time of maximum plasma concentration); AUC (area under the curve); T_half_ (plasma half time); MRT (mean residence time); Vz (Volume of distribution); Vss (Steady state volume of distribution); Clast (last observed plasma concentration); Tlast (Last time point with observable concentration).

| \| Parameter \| Dose \| Cmax \| Tmax \| AUClast \| \| AUCtot \| \| Extrapolated AUC \| \| T_half_ \| MRT \| \| Clearance \| Vz \| \| Vss \| \| --- \| --- \| --- \| --- \| --- \| --- \| --- \| --- \| --- \| --- \| --- \| --- \| --- \| --- \| --- \| --- \| --- \| \| Unit \| mg/kg \| ng/mL \| h \| ng/mL*h \| \| ng/mL*h \| \| % \| \| H \| H \| \| mL/min/kg \| L/kg \| \| L/kg \| \| **E5** \| 1 (IV) \| 1345 \| 0.08 \| 1875 \| 2312 \| \| 0.02 \| \| 5.0 \| \| 2.1 \| 7.21 \| \| 3.12 \| 0.90 \| \| \| **E7** \| 1 (IV) \| 3113 \| 0.08 \| 38577 \| 42312 \| \| 1.6 \| \| 13.6 \| \| 16.2 \| 0.39 \| \| 0.46 \| 0.38 \| \| \| **E5** \| 20(P) \| 230.3 \| 2 \| 3001 \| 3003 \| \| 0.1 \| \| 7.5 \| \| 8.5 \| 111.01 \| \| 72.5 \| 56.9 \| \| \| **E7** \| 20(P) \| 5340 \| 3 \| 143763 \| 146235 \| \| 1.7 \| \| 13.7 \| \| 20.8 \| 2.28 \| \| 2.7 \| 2.8 \| \| |
| --- | --- | --- | --- | --- | --- | --- | --- | --- | --- | --- | --- | --- | --- | --- | --- | --- | --- | --- | --- | --- | --- | --- | --- | --- | --- | --- | --- | --- | --- | --- | --- | --- | --- | --- | --- | --- | --- | --- | --- | --- | --- | --- | --- | --- | --- | --- | --- | --- | --- | --- | --- | --- | --- | --- | --- | --- | --- | --- | --- | --- | --- | --- | --- | --- | --- | --- | --- | --- | --- | --- | --- | --- | --- | --- | --- | --- | --- | --- | --- | --- | --- | --- | --- | --- | --- | --- | --- | --- | --- | --- | --- | --- | --- | --- | --- | --- | --- | --- | --- | --- | --- | --- |
