# Supplementary material for: Destabilizers of the thymidylate synthase homodimer accelerate its proteasomal degradation and inhibit cancer growth
Source: eLife. 2022 Dec 7;11:e73862. doi: 10.7554/eLife.73862 (PMC9831607; doi:10.7554/eLife.73862)
Supplement: Transparent reporting form [file elife-73862-transrepform1.pdf]

eLife
Westbrook Centre
Milton Road
Cambridge CB4 1YG, UK
P 01223 855340
W elifesciences.org
T @elife

eLife's transparent reporting form

We encourage authors to provide detailed information *within their submission* to facilitate the interpretation and replication of experiments. Authors can upload supporting documentation to indicate the use of appropriate reporting guidelines for health-related research (see EQUATOR Network), life science research (see the BioSharing Information Resource), or the ARRIVE guidelines for reporting work involving animal research. Where applicable, authors should refer to any relevant reporting standards documents in this form.

If you have any questions, please consult our Journal Policies and/or contact us: editorial@elifesciences.org.

Sample-size estimation

- You should state whether an appropriate sample size was computed when the study was being designed
- You should state the statistical method of sample size computation and any required assumptions
- If no explicit power analysis was used, you should describe how you decided what sample (replicate) size (number) to use

Please outline where this information can be found within the submission (e.g., sections or figure legends), or explain why this information doesn't apply to your submission:

The appropriate sample size was computed when the study was being designed in the case of animal studies and statistical details are reported in the main text pag. 52-55, Figure 8 and *Figure 8-figure supplement 1*.

Sample size
Sample size for the **In vivo experiments on orthotopic pancreatic cancer** was determined based on our previous experimental results with the effects of antimetabolites in pancreatic cancer cells, as published in Avan et al, Cancer Res 2013. For in vivo experiments, we utilized similar vehicle and treated cohorts (N=6) to reach similar statistical power, as based on the results of our previously published research (Giovannetti et al., JNCI 2014).
Sample size of Ovarian Cancer and Colorectal cancer cell lines was determined according to our experience and literature reporting similar experiments (Cardinale et al, PNAS 2011; Pelà et al, J Med Chem 2014).
Data exclusion. Data was not excluded.

Replicates

- You should report how often each experiment was performed
- You should include a definition of biological versus technical replication
- The data obtained should be provided and sufficient information should be provided to indicate the number of independent biological and/or technical replicates
- If you encountered any outliers, you should describe how these were handled
- Criteria for exclusion/inclusion of data should be clearly stated
- High-throughput sequence data should be uploaded before submission, with a private link for reviewers provided (these are available from both GEO and ArrayExpress)

Please outline where this information can be found within the submission (e.g., sections or figure legends), or explain why this information doesn't apply to your submission:

All experimental details and information about biological replicates can be found in the legend of Figures 5, 6, 7, 8 and figure supplements (Figure 8-figure supplement 1, Figure 8-figure supplement 2, Figure 7 - Figure supplement 1, Figure 7 - Figure supplement 3, Figure 6-figure supplement 4, Figure 6B-source data 1.
No outliers encountered.
No data were excluded.
No high-throughput sequence data were obtained.

Replication. In the **In vivo experiments on orthotopic pancreatic cancer**. The positive and negative controls (vehicle and 5-FU treatments) successfully reproduced the results found in our previous studies (Cardinale et al, PNAS 2011; Pelà et al, J Med Chem 2014). All cell culture data represents the results of at least three biologically independent experiments. All data derived from animal experiments likewise represent the results obtained from at least six biological independent animals.
Other details.
Randomization. Animals were randomized into groups with tumors of equivalent size prior to the beginning of the in vivo pharmacological studies.
Blinding. Investigators were not blinded to group allocation during data collection or analysis, as cages were clearly marked to indicate the presence of a potential chemical hazard (5-FU or or E7). T However, blinding is not relevant for the studies conducted here, as the data is collected exclusively in numeric form which is not readily subject to bias by subjective interpretation.

In the **In vivo experiments on xenografted ovarian cancer** Athymic Nude-Foxn1nu female mice were subcutaneously inoculated with 2×106 A2780 cells. Implanted xenotransplanted mice, chosen randomly, were subjected to **E7**, 5FU and vehicle treatment, as indicated in the materials and methods section; n = 4 mice per group. * **E7**-treatment is statistically different to control (vehicle) for the tumor volume at 6th and 7th day (t-test p<0.05). Error bars indicate SEM. **b,** quantitative analysis of the AUC of tumor growth kinetics in control, **E7** and 5FU-treated mice. Bars are representative of the mean ± SEM obtained from all mice treated (t-test, * p<0.05). **c,** survival curves (Kaplan Meier) showing the percentage of survival of xenotransplanted mice, placebo control versus **E7** and 5FU-treated mice (Log-rank (Mantel-Cox) test P value=0.0024; n=4).

eLife Sciences Publications, Ltd is a limited liability non-profit non-stock corporation incorporated in the State of Delaware, USA, with company number 5030732, and is registered in the UK with company number FC030576 and branch number BR015634 at the address Westbrook Centre, Milton Road, Cambridge CB4 1YG.

Statistical reporting

- Statistical analysis methods should be described and justified
- Raw data should be presented in figures whenever informative to do so (typically when N per group is less than 10)
- For each experiment, you should identify the statistical tests used, exact values of N, definitions of center, methods of multiple test correction, and dispersion and precision measures (e.g., mean, median, SD, SEM, confidence intervals; and, for the major substantive results, a measure of effect size (e.g., Pearson's r, Cohen's d)
- Report exact p-values wherever possible alongside the summary statistics and 95% confidence intervals. These should be reported for all key questions and not only when the p-value is less than 0.05.

Please outline where this information can be found within the submission (e.g., sections or figure legends), or explain why this information doesn’t apply to your submission:

eLife Sciences Publications, Ltd is a limited liability non-profit non-stock corporation incorporated in the State of Delaware, USA, with company number 5030732, and is registered in the UK with company number FC030576 and branch number BR015634 at the address Westbrook Centre, Milton Road, Cambridge CB4 1YG.

Each experiment described in the main text and in the Supplementary Information and all Additional files report the statistical analysis used (see above).

All experimental details and information about biological replicates can be found in the legend of Figures 5, 6, 7, 8 and figure supplements (Figure 8-figure supplement 1, Figure 8-figure supplement 2, Figure 7 - Figure supplement 1, Figure 7 - Figure supplement 3, Figure 6-figure supplement 4, Figure 6B-source data 1.

Data Collection:
Molecular modelling experiments: The protein and ligand preparation were carried out with the SYBYL 8 (Tripos Inc., St. Louis, MO) modeling package. Dimeric forms of the inactive enzyme structures were obtained from the PISA server and the Protein Quaternary file server. Molecular dynamics simulations were carried out with the AMBER 8 simulation package. PASS, SiteID (Tripos Inc., St. Louis, MO) and CASTp were used to identify the interface and surface pockets and CASTp server was used for calculation of the surface pocket sizes. The GRID software (Molecular Discovery) was used to calculate molecular interaction fields. The KYG server was used to predict the interface residues that could bind to RNA.

Data analysis Molecular modelling: Visual structure analysis was conducted using SYBYL 7.3 and PyMOL (version 1.8).
Statistical analysis was conducted using Prism 6/7/8 (GraphPad Software). (version 3.5.0). SPSS version 25 (SPSS, Inc., USA) and Prism 5.03 (GraphPad Software).
For pharmacokinetic data and FRET data: The statistical analysis was performed using unpaired t-test using Prism 8 for windows (version 3.1.1).

Cancer cells biological data were analyzed with one-way ANOVA whit the statistical software SPSS version 25 (SPSS, Inc., USA), Prism 5 version 5.03 and Prism 8 version 3.1.1 (GraphPad Software), where indicated. Selected pairwise comparisons were made using Tukey post-hoc analysis. Additional information is provided in the Supplementary Information and in each legend of main text figures.

The following information are given or in the Figure legends, or in the Supplementary Information for each experiment reported in the upoloaded files.
The exact sample size (n) for each experimental group/condition, is given as a discrete number and unit of measurement
A statement on whether measurements were taken from distinct samples or whether the same sample was measured repeatedly.
The statistical test(s) used AND whether they are one- or two-sided
A description of all covariates tested is given
A full description of the statistical parameters including central tendency (e.g. means) or other basic estimates (e.g. regression coefficient) AND variation (e.g. standard deviation) or associated estimates of uncertainty (e.g. confidence intervals)
For null hypothesis testing, the test statistic (e.g. F, t, r) with confidence intervals, effect sizes, degrees of freedom and P value noted
Give P values as exact values whenever suitable.

Group allocation

- Indicate how samples were allocated into experimental groups (in the case of clinical studies, please specify allocation to treatment method); if randomization was used, please also state if restricted randomization was applied
- Indicate if masking was used during group allocation, data collection and/or data analysis

This case does not apply.

Please outline where this information can be found within the submission (e.g., sections or figure legends), or explain why this information doesn't apply to your submission:

Additional data files ("source data")

- We encourage you to upload relevant additional data files, such as numerical data that are represented as a graph in a figure, or as a summary table
- Where provided, these should be in the most useful format, and they can be uploaded as "Source data" files linked to a main figure or table
- Include model definition files including the full list of parameters used
- Include code used for data analysis (e.g., R, MatLab)
- 2.69, 10.92 Avoid stating that data files are "available upon request"

Please indicate the figures or tables for which source data files have been provided:
Source Data files are available are accessible to Editors and Reviewers and are uploaded in the website.

Figure 1 contains the following associated file.

Figure 1 - Figure supplement 1. Y202 and K47 pockets at the hTS monomer-monomer interface (PDB ID: 1HVY, A chain)

Figure 2 contains the following associated file.

Figure 2 - Figure supplement 1. X-ray crystallographic structure of hTS-Y202C and hTS C195S-Y202C

Figure 3 contains the following associated files

Figure 3-figure supplement 1. Anion-exchange chromatograms of hTS obtained with a 280 nm and 350 nm detection

Figure 3-figure supplement 1-Source data file 1. Anion-exchange chromatograms of hTS obtained with a 280 nm and 350 nm detection

Figure 3-figure supplement 2. Concentration profile of compound C3 in the AE chromatographic fractions determined by high-resolution MS (UltiMate 3000 UHPLC tandem Orbitrap Q-Ex).

Figure 3-figure supplement 2-Source data 1. *Concentration profile* of compound C3 in the AE chromatographic fractions determined by high-resolution MS (UltiMate 3000 UHPLC tandem Orbitrap Q-Ex).

Figure 4 contains the following associated files.

Figure 4-Source data 1. Spectroscopic and mechanistic analysis of hTS dissociative inhibition for compounds C2, C3 and C4.

Figure 4F-Source data 1. Inhibition of hTS by compound C3.

Figure 6 contains the following associated files:

Figure 6B-Source data 1. Quantification of Annexin V-positive cells of apoptosis of A2780 and A2780/CP cells treated with E7 or PMX

Figure 6-figure supplement 1. TS mRNA expression in the primary pancreatic cancer cells.

eLife Sciences Publications, Ltd is a limited liability non-profit non-stock corporation incorporated in the State of Delaware, USA, with company number 5030732, and is registered in the UK with company number FC030576 and branch number BR015634 at the address Westbrook Centre, Milton Road, Cambridge CB4 1YG.

Figure 6-figure supplement 1-Source data 1. TS mRNA expression in the primary pancreatic cancer cells.

Figure 6–figure supplement 2. Pancreatic cancer cells LPc167 growth inhibition for compounds E7, E5 and 5FU.
Figure 6–figure supplement 2-Source data 1. Pancreatic cancer cells LPc167 growth inhibition for compounds E7, E5 and 5FU.

Figure 6-figure supplement 3. Ovarian cancer cell growth inhibition
Figure 6-figure supplement 3-Source data 1. Ovarian cancer cell growth inhibition

Figure 6-figure supplement 4. Colon cancer cell growth inhibition
Figure 6-figure supplement 4-Source data 1. Colon cancer cell growth inhibition

Figure 7 contains the following associated files

Figure 7-Source data 1. Effect of Dimer disrupters and hTS levels and half-life in human cancer cell lines (Figure C, D, G).
Figure 7-Source data 2. Western blot data of dimer disrupters effect on hTS levels in human cancer cell lines.

Figure 7 - figure supplement 1. Effect of cisplatin on cell death, and effect of dimer disrupters on hTS activity and hTS protein levels in cells.
Figure 7-figure supplement 1a,b-Source data 1**.** Flow cytometric analysis of apoptosis of A2780 and A2780/CP cells treated with E7, PMX and cisplatin.
Figure 7-figure supplement 1c,d- Source data 1. Effect of dimer disrupters on hTS protein levels in HT29 cells, WB analysis.
Figure 7-figure supplement 1e,f- Source data 1. TS activity assay for A2780 and A2780/CP cell lines after treatment with E5 and E7.
Figure 7 - figure supplement 2. Example of gating strategy used for cell analysis.
Figure 7 - figure supplement 3. Expression levels of total (exogenous plus endogenous) and exogenous hTS mRNAs in HCT116 cells with wild-type hTS-Myc-DDK tagged or mutant hTS-Myc-DDK-F59A vectors (in Figure 7G).

Figure 8 contains the following associated file.

Figure 8-Source data 1. Orthotopic pancreatic cancer in vivo.
Figure 8 - figure supplement 1. In vivo effects of E7 on Athymic Nude-Foxn1nu female mice
Figure 8-figure supplement 1-Source data 1. *In vivo effects of E7 on Athymic Nude-Foxn1nu female mi*
Figure 8 - figure supplement 2. Real time PCR (qPCR). Quantitative PCR (qPCR) measurements of hTS transcripts in A2780 and A2780/CP cells treated with E7, E5 and 5FU.
Figure 8 - figure supplement 2-Source data 1. Real time PCR (qPCR). Quantitative PCR (qPCR) measurements of hTS transcripts in A2780 and A2780/CP cells treated with E7, E5 and 5FU**.**
Figure 8-figure supplement 3. Representative immunohistochemical images showing extensive caspase-3 activation after treatment with E7

eLife Sciences Publications, Ltd is a limited liability non-profit non-stock corporation incorporated in the State of Delaware, USA, with company number 5030732, and is registered in the UK with company number FC030576 and branch number BR015634 at the address Westbrook Centre, Milton Road, Cambridge CB4 1YG.

STRUCTURAL DATA

Protein Data Bank (PDB) structures used in this study are found with the ID codes: 1JU6, 3H9K, 1HVY, 1YPV, 3N5G, 2RDA. Crystal structures determined in this study have been deposited to the PDB with the codes: 4O1U and 4O1X.

1. 4O1U, Crystal structure of human thymidylate synthase mutant Y202C (hTS-Y202C) obtained at 2.26 Å resolution, in the space group $P3_1$. The hTS-Y202C mutant was crystallized under high salt conditions using the vapor diffusion technique in hanging drop, at the temperature of 300 K. DOI: 10.2210/pdb4O1U/pdb. Available at https://www.rcsb.org/structure/4O1U

2.4O1X, Crystal structure of human thymidylate synthase double mutant C195S-Y202C, obtained at 2.32 Å resolution, in the space group $P2_12_12_1$. The double mutant hTS C195S-Y202C was crystallized under high salt conditions using the vapor diffusion technique in hanging drop, at the temperature of 300 K. DOI: 10.2210/pdb4O1X/pdb. Available at https://www.rcsb.org/structure/4O1X

Sequence of C195S-Y202C is reported in the Supplementary Information pg.4

STRUCTURAL DATA

Protein Data Bank (PDB) structures used in this study are found with the ID codes: 1JU6, 3H9K, 1HVY, 1YPV, 3N5G, 2RDA. Crystal structures determined in this study have been deposited to the PDB with the codes: 4O1U and 4O1X.

1. 4O1U, Crystal structure of human thymidylate synthase mutant Y202C (hTS-Y202C) obtained at 2.26 Å resolution, in the space group $P3_1$. The hTS-Y202C mutant was crystallized under high salt conditions using the vapor diffusion technique in hanging drop, at the temperature of 300 K. DOI: 10.2210/pdb4O1U/pdb. Available at https://www.rcsb.org/structure/4O1U

2.4O1X, Crystal structure of human thymidylate synthase double mutant C195S-Y202C, obtained at 2.32 Å resolution, in the space group $P2_12_12_1$. The double mutant hTS C195S-Y202C was crystallized under high salt conditions using the vapor diffusion technique in hanging drop, at the temperature of 300 K. DOI: 10.2210/pdb4O1X/pdb. Available at https://www.rcsb.org/structure/4O1X

Sequence of C195S-Y202C is reported in the Supplementary Information pg.4

eLife Sciences Publications, Ltd is a limited liability non-profit non-stock corporation incorporated in the State of Delaware, USA, with company number 5030732, and is registered in the UK with company number FC030576 and branch number BR015634 at the address Westbrook Centre, Milton Road, Cambridge CB4 1YG.
